# Supplementary material for: Dental topography and diet in marsupials and comparisons with primates
Source: J Mamm Evol. 2026 May 4;33(2):18. doi: 10.1007/s10914-025-09798-9 (PMC13139304; doi:10.1007/s10914-025-09798-9)
Supplement: Supplementary file 2 — Supplementary Material 2 [file 10914_2025_9798_MOESM2_ESM.pdf]

Journal of Mammalian Evolution: Dental topography and diet in marsupials and comparisons with primates

Dorien de Vries[*,1,2,3,a] Amy Gao[*,4], Darin A. Croft[4], Garrett Brown[4], Nicholas Burkart[4], April Connell[1], Ellie Gahan[1], Emily Spinks[1], Robin M.D. Beck[1]

[*] *shared first author*
[1] *School of Science, Engineering and Environment, University of Salford, Salford, UK*
[2] *Division of Integrative Anatomical Sciences, University of Southern California, Los Angeles, CA, USA*
[3] *Naturalis Biodiversity Center, Leiden, the Netherlands*
[4] *Department of Anatomy, Case Western Reserve University, Cleveland, OH, USA*
[a] *corresponding author, dorien.devries@naturalis.nl*

Online Resource 2

Content **page**

Table S1: Paired t-test/Wilcoxon signed-rank test *p*-values of m2 vs. m3 marsupial data 1
Full results of paired t-test (*t*-values, df, *p*-values) of m2-only vs. m3-only marsupial data 2
ANOVA & Tukey HSD or Kurskal-Wallis & Dunn test results of marsupial data 3
m2-only 3
m3-only 5
total marsupial sample (m2 & m3) 7
Fig. S1. PCA plot of marsupial sample for ariaDNE, RFI, lnOA, TriTaHI, and OPCR 11
Fig. S2. PCA plots for marsupial m2-only sample and m3-only sample 13
Full results of Welch t-test/Wilcoxon rank-sum test of marsupial versus primate specimen data 14
Fig. S3. Boxplots of marsupial and primate primate data with individual data points 15
Fig. S4. Marsupial insectivore molar shape variation 16
Table S2. Leave-one-out QDAs results based on <u>specimen values</u> vs. [*species averages*] 17
QDA results: combined sample classification accuracy per dietary category 18
Table S3. Exact same marsupial sample: m2-only versus m3-only versus m2&m3 comparison 20
Table S4. QDA results (marsupial = training set, primates = test set) of <u>specimen values</u> vs. [*species averages*] 21
Table S5. QDA category specific results (marsupials m2 = training set, primates = test set) 22
Table S6. QDA category specific results (marsupials m3 = training set, primates = test set) 22
Table S7. QDA results (primates = training set, marsupials = test set) of <u>specimen values</u> vs. [*species averages*] 23
Fig. S5. Sensitivity of curvature metrics to material. 25
Fig. S6. 2D-visualisation of QDA for <u>marsupial</u> (m2&m3, m2-only, m3-only) 26
Fig. S7. 2D-visualisation of QDA for <u>primate</u> 27
Fig. S8. 2D-visualisation of QDA for <u>marsupial (m2 & m3) and primate</u> 28
Fig. S9. PCA plot of total sample (Fig. 7) with primate data labeled 29
Table S8. MorphoSource data information and funding sources 30
Justification of diets 34

Table S1. The *p* values of paired t-test or, if one or both groups had non-normally distributed data, Wilcoxon signed-rank test (underlined) of m2-only vs. m3-only. Om = omnivore; Fr-In = frugivore-insectivore; In = insectivore; Fau = faunivore; and Fo = folivore. Significant ($p < 0.05$) results are **bolded**. Frugivores were excluded due to small sample size.

Metric	Om	Fr-In	In	Fau	Fo
ariaDNE	0.871	0.158	0.196	0.096	**0.014**
RFI	0.495	0.938	0.847	0.146	0.255
ln(OA)	0.890	0.560	**0.041**	**0.007**	0.424
Trigonid-Talonid RI	0.263	0.125	0.148	0.189	**0.017**
OPCR	0.994	0.551	0.359	0.647	0.781
ariaDNE CV	0.531	0.577	0.365	1.0	0.119

Full results of paired t-test (*t*-values, df, and *p*-values) or, if one or both groups had non-normally distributed data, <u>Wilcoxon signed-rank test</u> (*V* values and *p*-values, underlined) of m2-only vs. m3-only marsupial datasets. Significant *p*-values (<0.05) are bolded.

**ariaDNE**	t / <u>V</u>	df	*p*-value
Folivory	-2.8282	13	**0.01424**
Insectivory	-1.3854	10	0.1961
Frugivory-Insectivory	1.8721	3	0.1579
Faunivory	-2.164	4	0.09645
Omnivory	-0.20535	1	0.8711

**RFI**	t / <u>V</u>	df	*p*-value
Folivory	-1.1915	13	0.2547
Insectivory	-0.19788	10	0.8471
Frugivory-Insectivory	-0.083983	3	0.9384
Faunivory	-1.803	4	0.1457
Omnivory	1.0153	1	0.4952

**lnOA**	t / <u>V</u>	df	*p*-value
Folivory	-0.82613	13	0.4236
Insectivory	2.3499	10	**0.04065**
Frugivory-Insectivory	0.65281	3	0.5604
Faunivory	-5.1398	4	**0.006792**
Omnivory	0.17385	1	0.8904

**TriTaHI**	t / <u>V</u>	df	*p*-value
Folivory	-2.7194	13	**0.01753**
Insectivory	<u>16</u>		<u>0.1475</u>
Frugivory-Insectivory	<u>0</u>		<u>0.125</u>
Faunivory	-1.5806	4	0.1891
Omnivory	-2.2848	1	0.2626

**OPCR**	t / <u>V</u>	df	*p*-value
Folivory	-0.28446	13	0.7805
Insectivory	-0.96208	10	0.3587
Frugivory-Insectivory	0.67002	3	0.5508
Faunivory	-0.49395	4	0.6472
Omnivory	0.0091463	1	0.9942

**ariaDNE CV**	t / <u>V</u>	df	*p*-value
Folivory	<u>78</u>		<u>0.1189</u>
Insectivory	<u>44</u>		<u>0.3652</u>
Frugivory-Insectivory	0.62363	3	0.5771
Faunivory	<u>8</u>		<u>1</u>
Omnivory	-0.90833	1	0.5306

ANOVA & Tukey HSD or Kurskal-Wallis & Dunn test results of marsupial data (m2-only, m3-only, m2&m3 combined).
As mentioned in the main text, the frugivore category was dismissed from these results as the sample size was only 2 (one marsupial m2, one marsupial m3).

Marsupial m2 only dataset

ariaDNE (ANOVA & Tukey HSD)

ANOVA:

	Df	Sum Sq	Mean Sq	F value	Pr(>F)
diet	5	0.001426	0.0002852	2.713	**0.0357 ***
Residuals	35	0.003679	0.0001051		

Shapiro-Wilk normality test:
W = 0.97714, p-value = 0.569

Tukey HSD:

	diff	lwr	upr	p adj
Folivory-Faunivory	-0.0074503701	-0.023166406	0.008265666	0.7099012
Frugivory-Faunivory	-0.0273579082	-0.061198017	0.006482201	0.1718424
Frugivory-Insectivory-Faunivory	0.0053114614	-0.014226135	0.024849057	0.9619973
Insectivory-Faunivory	-0.0003943466	-0.017056064	0.016267371	0.9999997
Omnivory-Faunivory	0.0009984318	-0.024847378	0.026844242	0.9999967
Frugivory-Folivory	-0.0199075381	-0.051694786	0.011879709	0.4265629
Frugivory-Insectivory-Folivory	0.0127618315	-0.002954204	0.028477867	0.1683197
Insectivory-Folivory	0.0070560236	-0.004897594	0.019009641	0.4918889
Omnivory-Folivory	0.0084488019	-0.014644098	0.031541702	0.8770473
Frugivory-Insectivory-Frugivory	0.0326693696	-0.001170739	0.066509479	0.0635851
Insectivory-Frugivory	0.0269635616	-0.005301716	0.059228840	0.1464246
Omnivory-Frugivory	0.0283563400	-0.009478052	0.066190732	0.2381463
Insectivory-Frugivory-Insectivory	-0.0057058080	-0.022367526	0.010955910	0.9039144
Omnivory-Frugivory-Insectivory	-0.0043130296	-0.030158840	0.021532781	0.9957393
Omnivory-Insectivory	0.0013927784	-0.022353823	0.025139380	0.9999738

OPCR (ANOVA)

ANOVA:

	Df	Sum Sq	Mean Sq	F value	Pr(>F)
diet	5	1965	393.1	0.771	0.577
Residuals	35	17844	509.8		

Shapiro-Wilk normality test
W = 0.95058, p-value = 0.07334

RFI (ANOVA)

ANOVA:

	Df	Sum Sq	Mean Sq	F value	Pr(>F)
diet	5	0.07337	0.014674	1.99	0.104
Residuals	35	0.25808	0.007374		

Shapiro-Wilk normality test:

W = 0.97955, p-value = 0.6583

ariaDNE CV (ANOVA)
ANOVA**:**

	Df	Sum Sq	Mean Sq	F value	Pr(>F)
diet	5	0.0298	0.005954	0.601	0.7
Residuals	35	0.3469	0.009911		

Shapiro-Wilk normality test:
W = 0.954, p-value = 0.09677

lnOA (ANOVA & Tukey HSD)
ANOVA:

	Df	Sum Sq	Mean Sq	F value	Pr(>F)
diet	5	25.93	5.187	14.65	**9.29e-08** ***
Residuals	35	12.39	0.354		

Shapiro-Wilk normality test:
W = 0.95485, p-value = 0.1037

Tukey HSD:

	diff	lwr	upr	p adj
Folivory-Faunivory	0.63879984	-0.2734506	1.551050266	0.3057310
Frugivory-Faunivory	-0.25858769	-2.2228651	1.705689701	0.9986188
Frugivory-Insectivory-Faunivory	-1.14364528	-2.2777214	-0.009569192	**0.0471049**
Insectivory-Faunivory	-1.12041676	-2.0875601	-0.153273427	**0.0153714**
Omnivory-Faunivory	-0.20698404	-1.7072257	1.293257601	0.9982705
Frugivory-Folivory	-0.89738754	-2.7425049	0.947729792	0.6875989
Frugivory-Insectivory-Folivory	-1.78244512	-2.6946955	-0.870194695	**0.0000153**
Insectivory-Folivory	-1.75921660	-2.4530743	-1.065358909	**0.0000001**
Omnivory-Folivory	-0.84578388	-2.1862305	0.494662779	0.4182982
Frugivory-Insectivory-Frugivory	-0.88505758	-2.8493350	1.079219814	0.7510983
Insectivory-Frugivory	-0.86182907	-2.7346941	1.011035946	0.7345244
Omnivory-Frugivory	0.05160365	-2.1445252	2.247732545	0.9999997
Insectivory-Frugivory-Insectivory	0.02322852	-0.9439148	0.990371849	0.9999997
Omnivory-Frugivory-Insectivory	0.93666123	-0.5635804	2.436902876	0.4299856
Omnivory-Insectivory	0.91343272	-0.4649586	2.291824045	0.3644611

TriTaHI (ANOVA & Tukey HSD)
ANOVA:

	Df	Sum Sq	Mean Sq	F value	Pr(>F)
diet	5	0.4683	0.09366	7.142	**0.000107** ***
Residuals	35	0.4590	0.01311		

Shapiro-Wilk normality test:
W = 0.95338, p-value = 0.09203

Tukey HSD:

	diff	lwr	upr	p adj
Folivory-Faunivory	-0.28966193	-0.46521157	-0.11411229	**0.0002370**
Frugivory-Faunivory	-0.20726521	-0.58526250	0.17073208	0.5710047
Frugivory-Insectivory-Faunivory	-0.07236906	-0.29060590	0.14586778	0.9150657
Insectivory-Faunivory	-0.11620798	-0.30232098	0.06990502	0.4298890
Omnivory-Faunivory	-0.17285874	-0.46155893	0.11584146	0.4762449
Frugivory-Folivory	0.08239672	-0.27266991	0.43746334	0.9808085
Frugivory-Insectivory-Folivory	0.21729287	0.04174323	0.39284251	**0.0081833**
Insectivory-Folivory	0.17345395	0.03993089	0.30697701	**0.0049549**
Omnivory-Folivory	0.11680319	-0.14114673	0.37475312	0.7472655
Frugivory-Insectivory-Frugivory	0.13489615	-0.24310114	0.51289344	0.8878121
Insectivory-Frugivory	0.09105723	-0.26934904	0.45146351	0.9721899
Omnivory-Frugivory	0.03440647	-0.38820734	0.45702029	0.9998672
Insectivory-Frugivory-Insectivory	-0.04383892	-0.22995192	0.14227408	0.9795044
Omnivory-Frugivory-Insectivory	-0.10048968	-0.38918987	0.18821052	0.8978041
Omnivory-Insectivory	-0.05665076	-0.32190259	0.20860108	0.9867433

Marsupial m3 only dataset

ariaDNE (ANOVA)

ANOVA:

	Df	Sum Sq	Mean Sq	F value	Pr(>F)
diet	5	0.001258	0.0002516	2.132	0.0853
Residuals	34	0.004012	0.0001180		

Shapiro-Wilk normality test

W = 0.97215, p-value = 0.4198

OPCR (Kruskal-Wallis)

ANOVA:

	Df	Sum Sq	Mean Sq	F value	Pr(>F)
diet	5	3668	733.6	1.532	0.206
Residuals	34	16285	479.0		

Shapiro-Wilk normality test

W = 0.92327, p-value = **0.009772**

Kruskal-Wallis rank sum test

data: OPCR by diet

Kruskal-Wallis chi-squared = 7.4396, df = 5, p-value = 0.1899

RFI (ANOVA)

ANOVA:

	Df	Sum Sq	Mean Sq	F value	Pr(>F)
diet	5	0.08672	0.017345	2.051	0.0961
Residuals	34	0.28753	0.008457		

Shapiro-Wilk normality test

W = 0.98288, p-value = 0.7943

ariaDNE CV (Kruskal-Wallis)
ANOVA:

	Df	Sum Sq	Mean Sq	F value	Pr(>F)
diet	5	0.1225	0.024491	2.572	**0.0446 ***
Residuals	34	0.3237	0.009522		

Shapiro-Wilk normality test:
W = 0.93972, p-value = **0.03384**

Kruskal-Wallis rank sum test:
Kruskal-Wallis chi-squared = 7.6309, df = 5, p-value = 0.1778

lnOA (ANOVA & TukeyHSD)
ANOVA:

	Df	Sum Sq	Mean Sq	F value	Pr(>F)
diet	5	26.89	5.377	15.49	**6.06e-08 *****
Residuals	34	11.80	0.347		

Shapiro-Wilk normality test:
W = 0.95573, p-value = 0.1195

Tukey HSD:

	diff	lwr	upr	p adj
Folivory-Faunivory	0.5218933	-0.3371654	1.3809519	0.4587785
Frugivory-Faunivory	-0.5169776	-2.4378912	1.4039359	0.9632658
Frugivory-Insectivory-Faunivory	-1.4069308	-2.5548962	-0.2589654	**0.0091083**
Insectivory-Faunivory	-1.3153835	-2.2179660	-0.4128010	**0.0013241**
Omnivory-Faunivory	-0.3211818	-1.5787149	0.9363513	0.9706033
Frugivory-Folivory	-1.0388709	-2.8756156	0.7978738	0.5366929
Frugivory-Insectivory-Folivory	-1.9288240	-2.9295971	-0.9280510	**0.0000208**
Insectivory-Folivory	-1.8372767	-2.5432352	-1.1313183	**0.0000001**
Omnivory-Folivory	-0.8430751	-1.9678469	0.2816967	0.2372469
Frugivory-Insectivory-Frugivory	-0.8899531	-2.8782876	1.0983813	0.7548982
Insectivory-Frugivory	-0.7984058	-2.6559054	1.0590937	0.7843843
Omnivory-Frugivory	0.1957958	-1.8577472	2.2493388	0.9997084
Insectivory-Frugivory-Insectivory	0.0915473	-0.9468265	1.1299211	0.9998014
Omnivory-Frugivory-Insectivory	1.0857490	-0.2725421	2.4440400	0.1804676
Omnivory-Insectivory	0.9942017	-0.1641528	2.1525561	0.1273301

TriTaHI (Kruskal-Wallis & Dunn test)
ANOVA:

	Df	Sum Sq	Mean Sq	F value	Pr(>F)
diet	5	0.5551	0.11101	8.49	**2.7e-05 *****
Residuals	34	0.4446	0.01308		

Shapiro-Wilk normality test
W = 0.94459, p-value = **0.04948**

Kruskal-Wallis rank sum test:
Kruskal-Wallis chi-squared = 18.783, df = 5, p-value = **0.002109**

Dunn tests: Comparison of x by group (Dunn test)

Col Mean-Row Mean	Faunivor	Folivory	Frugivor	Fru-Ins	Insectiv
Folivory	3.736480 **0.0014***				
Frugivor	0.884339 1.0000	-0.822714 1.0000			
Fru-Ins	0.353380 1.0000	-2.802021 **0.0381**	-0.650328 1.0000		
Insectiv	1.590963 0.8371	-2.512725 0.0899	-0.141460 1.0000	0.992231 1.0000	
Omnivory	0.987935 1.0000	-1.749239 0.6019	-0.222239 1.0000	0.615989 1.0000	-0.167146 1.0000

Total marsupial dataset (m2 & m3)

ariaDNE (ANOVA and Tukey HSD)

ANOVA:

	Df	Sum Sq	Mean Sq	F value	Pr(>F)
diet	5	0.002656	0.0005311	5.098	**0.000446** ***
Residuals	75	0.007814	0.0001042		

Shapiro-Wilk normality test:
W = 0.97986, p-value = 0.2321

Tukey HSD:

	diff	lwr	upr	p adj
Folivory-Faunivory	-0.008823381	-0.019257099	0.001610338	0.1453642
Frugivory-Faunivory	-0.029362962	-0.0523105239	-0.006415400	**0.0046177**
Frugivory-Insectivory-Faunivory	0.002196431	-0.0112211312	0.015613993	0.9967657
Insectivory-Faunivory	-0.002333852	-0.0133575038	0.008689801	0.9892921
Omnivory-Faunivory	-0.000196325	-0.0162973997	0.015904750	1.0000000
Frugivory-Folivory	-0.020539581	-0.0422979175	0.001218756	0.0753665
Frugivory-Insectivory-Folivory	0.011019812	-0.0002436502	0.022283274	0.0587595
Insectivory-Folivory	0.006489529	-0.0017782099	0.014757268	0.2088132
Omnivory-Folivory	0.008627056	-0.0057284034	0.022982515	0.4989767
Frugivory-Insectivory-Frugivory	0.031559393	0.0082228614	0.054895924	**0.0022973**
Insectivory-Frugivory	0.027029110	0.0049818058	0.049076415	**0.0075784**
Omnivory-Frugivory	0.029166637	0.0041905015	0.054142772	**0.0127252**
Insectivory-Frugivory-Insectivory	-0.004530283	-0.0163423107	0.007281745	0.8708792
Omnivory-Frugivory-Insectivory	-0.002392756	-0.0190435129	0.014258001	0.9982615
Omnivory-Insectivory	0.002137527	-0.0126522550	0.016927308	0.9982132

OPCR (Kruskal-Wallis)

ANOVA:

	Df	Sum Sq	Mean Sq	F value	Pr(>F)
diet	5	5357	1071.4	2.336	**0.0501**
Residuals	75	34406	458.7		

Shapiro-Wilk normality test:
W = 0.95264, p-value = **0.004566**

Kruskal-Wallis rank sum test:
Kruskal-Wallis chi-squared = 8.9497, df = 5, p-value = 0.1111

RFI (ANOVA & Tukey HSD)
ANOVA:

	Df	Sum Sq	Mean Sq	F value	Pr(>F)
diet	5	0.1600	0.0320	4.386	**0.00148** **
Residuals	75	0.5471	0.0073		

Shapiro-Wilk normality test:
W = 0.98783, p-value = 0.6449

Tukey HSD:

	diff	lwr	upr	p adj
Folivory-Faunivory	-0.10877431	-0.196082801	-0.02146582	**0.0063175**
Frugivory-Faunivory	-0.13554736	-0.327570650	0.05647593	0.3169977
Frugivory-Insectivory-Faunivory	-0.05029902	-0.162576064	0.06197803	0.7785158
Insectivory-Faunivory	-0.09609924	-0.188344241	-0.00385423	**0.0362358**
Omnivory-Faunivory	0.01373361	-0.120998847	0.14846606	0.9996714
Frugivory-Folivory	-0.02677305	-0.208844997	0.15529890	0.9980591
Frugivory-Insectivory-Folivory	0.05847529	-0.035776420	0.15272701	0.4628608
Insectivory-Folivory	0.01267508	-0.056508677	0.08185883	0.9945080
Omnivory-Folivory	0.12250792	0.002382631	0.24263321	**0.0429838**
Frugivory-Insectivory-Frugivory	0.08524834	-0.110029815	0.28052650	0.7966348
Insectivory-Frugivory	0.03944812	-0.145041887	0.22393813	0.9887973
Omnivory-Frugivory	0.14928097	-0.059717256	0.35827919	0.3041847
Insectivory-Frugivory-Insectivory	-0.04580022	-0.144642285	0.05304185	0.7531623
Omnivory-Frugivory-Insectivory	0.06403263	-0.075299522	0.20336477	0.7595274
Omnivory-Insectivory	0.10983284	-0.013926819	0.23359250	0.1110764

ariaDNE CV (Kruskal-Wallis)
ANOVA:

	Df	Sum Sq	Mean Sq	F value	Pr(>F)
diet	5	0.1344	0.026871	2.927	**0.0181 ***
Residuals	75	0.6886	0.009181		

Shapiro-Wilk normality test
W = 0.96201, p-value = **0.01685**

Kruskal-Wallis rank sum test
Kruskal-Wallis chi-squared = 9.9473, df = 5, p-value = 0.07674

lnOA (Kruskal-Wallis & Dunn test)
ANOVA:

	Df	Sum Sq	Mean Sq	F value	Pr(>F)
diet	5	52.72	10.544	32.49	**<2e-16 *****
Residuals	75	24.34	0.325		

Shapiro-Wilk normality test:
W = 0.95862, p-value = **0.01041**

Kruskal-Wallis rank sum test:
Kruskal-Wallis chi-squared = 55.914, df = 5, p-value = **8.465e-11**

Dunn test: Comparison of x by group (Dunn test)

Col Mean-Row Mean	Faunivor	Folivory	Frugivor	Fru-Ins	Insectiv
Folivory	-1.578544<br>0.8583				
Frugivor	0.683639<br>1.0000	1.477959<br>1.0000			
Fru-Ins	2.965993<br>**0.0226***	4.995487<br>**0.0000***	1.033081<br>1.0000		
Insectiv	3.521148<br>**0.0032***	6.686954<br>**0.0000***	1.049019<br>1.0000	-0.083007<br>1.0000	
Omnivory	0.501496<br>1.0000	1.709782<br>0.6548	-0.304819<br>1.0000	-1.905124<br>0.4257	-2.078548<br>0.2824

TriTaHI (Kruskal-Wallis & Dunn test)
ANOVA:

	Df	Sum Sq	Mean Sq	F value	Pr(>F)
diet	5	1.0269	0.20538	16.42	**6.45e-11 *****
Residuals	75	0.9381	0.01251		

Shapiro-Wilk normality test:
W = 0.95913, p-value = **0.01118**

Kruskal-Wallis rank sum test:
Kruskal-Wallis chi-squared = 37.256, df = 5, p-value = **5.323e-07**

Dunn test: Comparison of x by group (Dunn test)

Col Mean-Row Mean	Faunivor	Folivory	Frugivor	Fru-Ins	Insectiv
Folivory	5.190643<br>**0.0000***				
Frugivor	1.032999<br>1.0000	-1.399596<br>1.0000			
Fru-Ins	0.910335<br>1.0000	-3.723831<br>**0.0015***	-0.492375<br>1.0000		

Insectiv	2.040486<br>0.3098	-3.829837<br>**0.0010***	-0.054936<br>1.0000	0.870226<br>1.0000	
Omnivory	1.243711<br>1.0000	-2.377675<br>0.1307	-0.147329<br>1.0000	0.469084<br>1.0000	-0.166907<br>1.0000

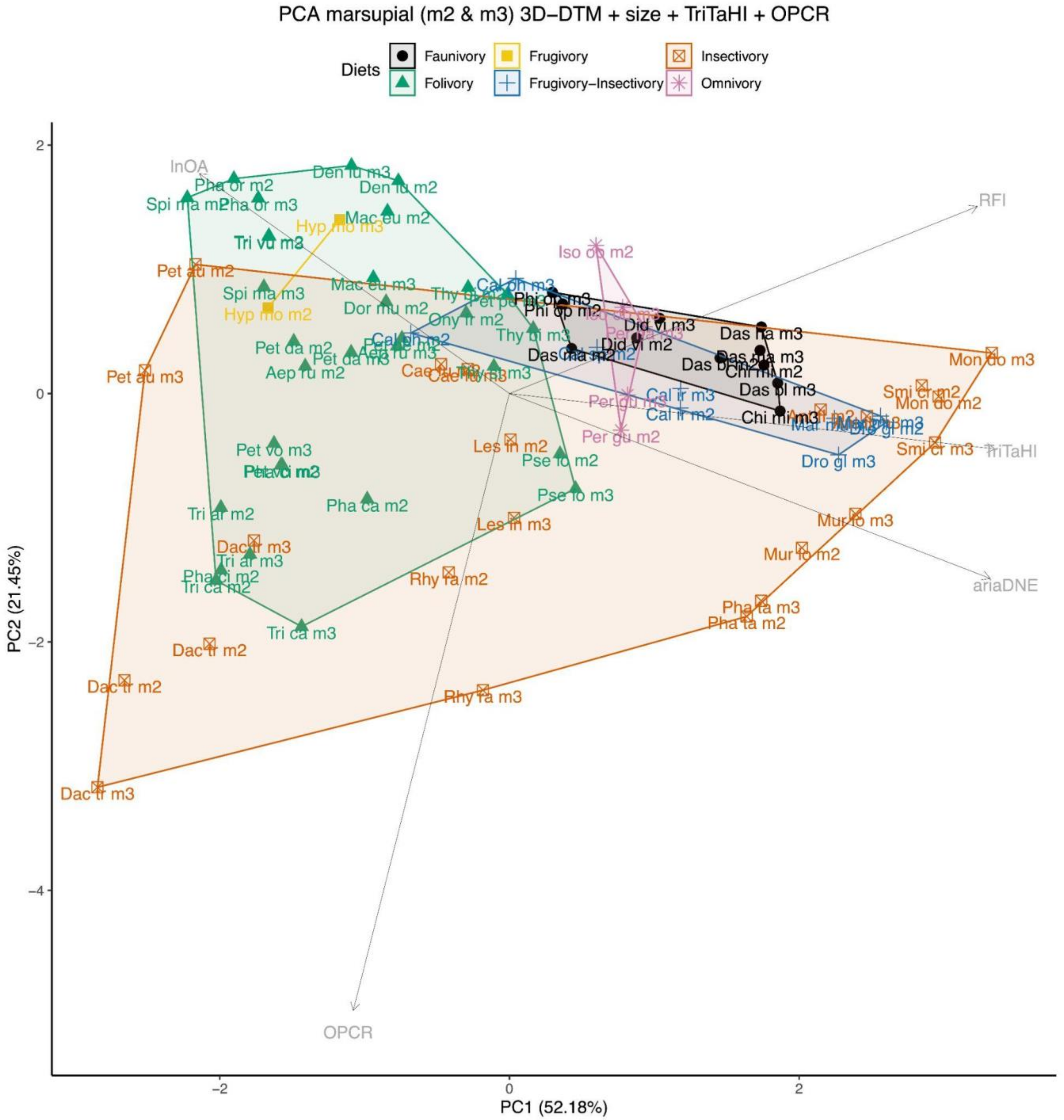

Fig. S1 Principal Component Analysis of 3D-DTM (= ariaDNE and RFI), lnOA, TriTaHI, and OPCR plot showing PC1 and PC2 scores for total marsupial sample, capturing 73.63% of the variation. Note that the PC loading arrows are not to scale.

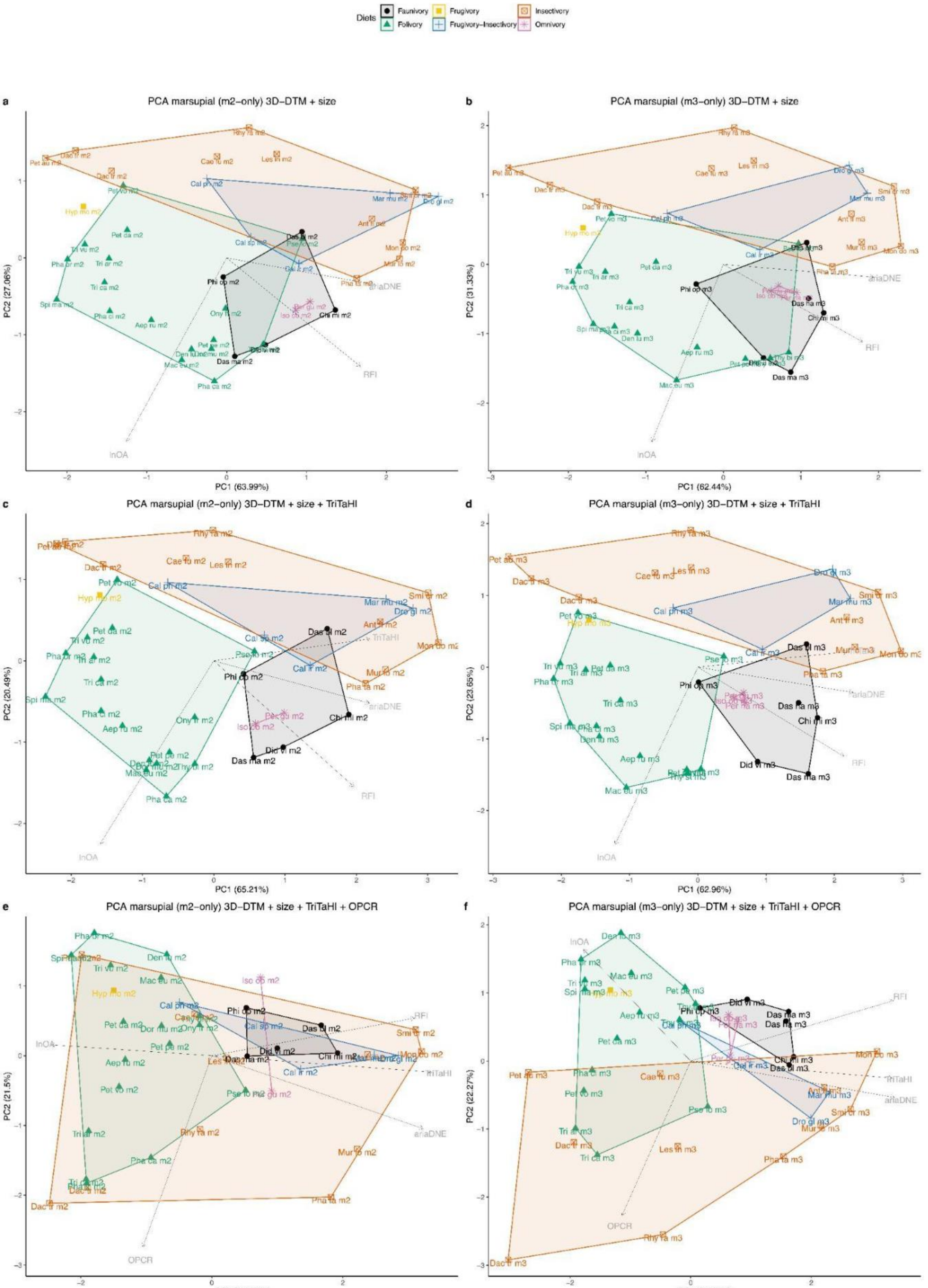

Diets
Faunivory
Folivory
Frugivory
Frugivory–Insectivory
Insectivory
Omnivory
a
PCA marsupial (m2–only) 3D–DTM + size
PC1 (63.99%)
PC2 (27.06%)
ariaDNE
RFI
lnOA
b
PCA marsupial (m3–only) 3D–DTM + size
PC1 (62.44%)
PC2 (31.33%)
ariaDNE
RFI
lnOA
c
PCA marsupial (m2–only) 3D–DTM + size + TriTaHI
PC1 (65.21%)
PC2 (20.49%)
TriTaHI
ariaDNE
RFI
lnOA
d
PCA marsupial (m3–only) 3D–DTM + size + TriTaHI
PC1 (62.96%)
PC2 (23.65%)
ariaDNE
RFI
lnOA
e
PCA marsupial (m2–only) 3D–DTM + size + TriTaHI + OPCR
PC1 (53.13%)
PC2 (21.5%)
lnOA
RFI
TriTaHI
ariaDNE
OPCR
f
PCA marsupial (m3–only) 3D–DTM + size + TriTaHI + OPCR
PC1 (51.44%)
PC2 (22.27%)
lnOA
RFI
TriTaHI
ariaDNE
OPCR

Fig. S2 Principal Component Analysis plots of marsupial m2-only (a, c, and e) and m3-only samples (b, d, and f) using 3D-DTM (= ariaDNE and RFI), lnOA (a and b), those metrics as well as TriTaHI (c and d), and finally all those metrics plus OPCR (e and f).

Full results of Welch t-test (*t*-values, df, and *p*-values) or, if one or both groups had non-normally distributed data, Wilcoxon signed-rank test (*W*-values and *p*-values, underlined) of marsupial (m2 & m3) vs. primate (platyrrhine & strepsirrhine) specimen-based datasets. Significant *p*-values (<0.05) are bolded. Note that results are listed for the frugivory category, but these were not included in the main manuscript as the marsupial frugivore sample was only *n* = 2.

**ariaDNE**	t / W	df	*p*-value
Folivory	250		**0.0003203**
Insectivory	52		**0.003131**
Frugivory-Insectivory	1.9366	8.7774	0.08558
Frugivory	-22.401	4.5413	**7.987e-06**

**RFI**	t / W	df	*p*-value
Folivory	419		0.2158
Insectivory	-1.354	28.498	0.1864
Frugivory-Insectivory	2.7299	9.549	**0.02205**
Frugivory	-2.6624	1.392	0.1684

**lnOA**	t / W	df	*p*-value
Folivory	313		**0.007084**
Insectivory	93		0.1677
Frugivory-Insectivory	183		0.2717
Frugivory	-6.4616	4.6025	**0.001796**

**TriTaHI**	t / W	df	*p*-value
Folivory	-8.292	61.996	**1.235e-11**
Insectivory	122		0.7358
Frugivory-Insectivory	426		**0.0001863**
Frugivory	4.1925	1.0991	0.1317

**OPCR**	t / W	df	*p*-value
Folivory	-8.9947	60.607	**9.233e-13**
Insectivory	20		**9.461e-06**
Frugivory-Insectivory	-22.276	51.328,	**< 2.2e-16**
Frugivory	-10.788	1.5434	**0.01945**

**ariaDNE CV**	t / W	df	*p*-value
Folivory	2		**< 2.2e-16**
Insectivory	-6.6215	24.971	**6.184e-07**
Frugivory-Insectivory	-5.9879	8.59	**0.0002476**
Frugivory	-34.37	3.964	**4.672e-06**

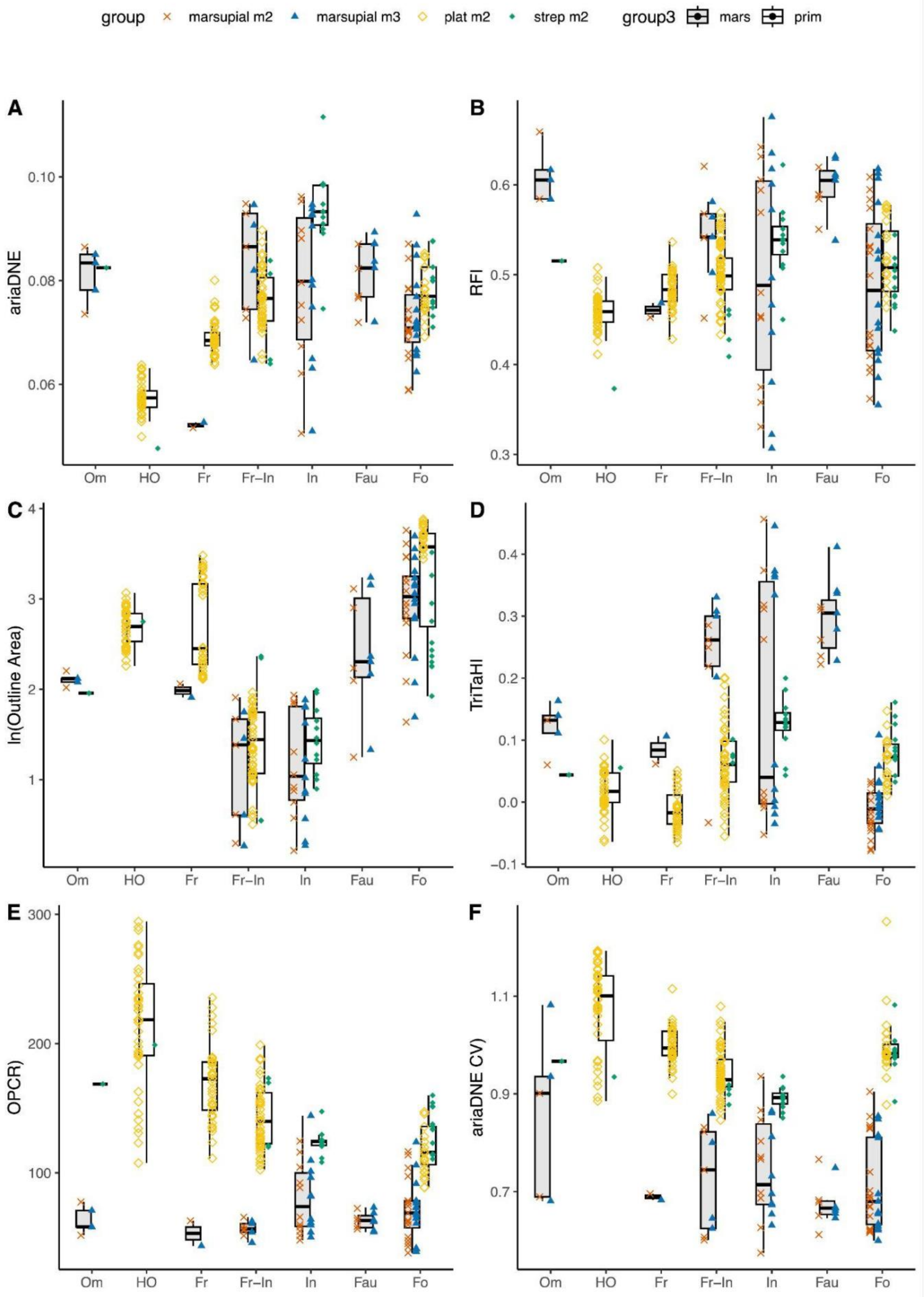

group
marsupial m2
marsupial m3
plat m2
strep m2
group3
mars
prim
A
ariaDNE
B
RFI
C
ln(Outline Area)
D
TriTaHI
E
OPCR)
F
ariaDNE CV)
Om
HO
Fr
Fr−In
In
Fau
Fo

Fig. S3 Boxplots of marsupial (m2 [orange cross] & m3 [blue triangle]) data in grey and primate (platyrrhine [yellow open diamonds] & strepsirrhine [green filled diamonds]) in white, both displaying specimen data distribution for different diets. All significantly different ($p < 0.05$) pairs are marked by brackets and asterisks. Om = omnivore; HO = hard-object feeder; Fr = frugivore; Fr-In = frugivore-insectivore; In = insectivore; Fau = faunivore; and Fo = folivore. * = $p < 0.05$; ** = $p < 0.01$; and *** = $p < 0.001$. Note that omnivores, hard-object feeders, frugivores, and faunivores were excluded from the t-tests due to small sample sizes.

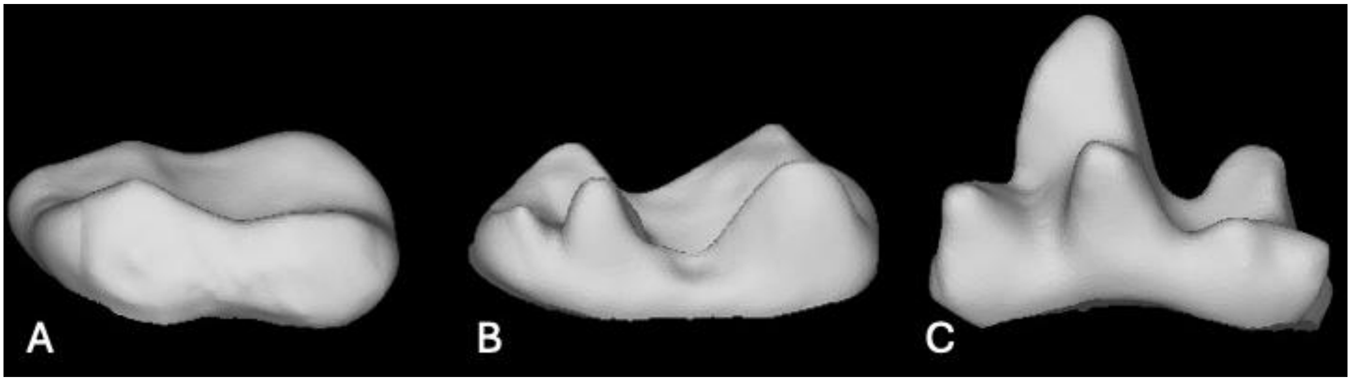

Fig. S4 Marsupial insectivore molar shape variation. A. *Petaurus australis* QM J8598 m2, member of ‘group 1’. B. *Lestoros inca* MVZ116049 m2, member of ‘group 2’. C. *Smithopsis crassicaudata* SAMA M12809 m2, member of ‘group 3’. See main text for information on grouping. Not to scale.

Table S2. Leave-one-out QDAs accuracy based on specimen values or [*species averages]*.

Metric	Marsupial m2s	Marsupial m3s	Marsupial all	Platyrrhines [*species averages only 3 diets, hence NA*]	Strepsirrhines [*species averages only 2 diets, hence NA*]	Primates [5]	Marsupial m2s + Primates [6]	Marsupial m3s + Primates [6]	Marsupial all + Primates [6]
RFI+ariaDNE	34.21% [*40.5%*]	55.56% [*57.1%*] (59.38%*) [*61.3%**]	51.35% [*46.2%*]	70.34% [*NA*]	71.43% (83.33%*) [*NA*]	62.64% [*62.7%*]	55.87% [*47.2%*]	61.14% [*52.9%*]	56.4% [*48.4%*]
RFI+ariaDNE+ size	55.26% [*51.4%*]	69.44% [*68.6%*] (78.13%*) [*77.4%**]	77.03% [*64.1%*]	97.24% [*NA*]	82.14% (95.83%*) [*NA*]	91.95% [*80.4%*]	79.34% [*60.7%*]	81.99% [*63.2%*]	79.6% [*64.8%*]
RFI+ariaDNE+ size+TrigTaHI	57.89% [*56.8%*]	87.5%* [*80.6%**]	79.73% [*69.2%*]	97.24% [*NA*]	91.67%* [*NA*]	91.38% [*74.5%*]	83.1% [*67.4%*]	82.46% [*70.1%*]	81.2% [*69.2%*]
RFI+ariaDNE+ size+ariaDNE CV	52.63% [*51.4%*]	81.25%* [*80.6%**]	78.39% [*66.7%*]	94.48% [*NA*]	91.67%* [*NA*]	88.51% [*76.5%*]	84.04% [*71.9%*]	86.73% [*80.5%*]	83.6% [*75.8%*]
RFI+ariaDNE+ size+OPCR	63.16% [*62.2%*]	78.13%* [*77.4%**]	79.73% [*61.5%*]	99.31% [*NA*]	91.67%* [*NA*]	93.10% [*74.5%*]	86.85% [*73.0%*]	88.15% [*78.2%*]	87.6% [*79.1%*]

Marsupial samples (m2 only, m3 only, and all): 4 different dietary categories (Folivory, Insectivory, Frugivory-Insectivory, Faunivory), unless marked with an * = Folivory, Insectivory, Faunivory)
Platyrrhine sample: 4 dietary categories (Folivory, Frugivory, Hard-Object feeder, Frugivory-Insectivory)
Strepsirrhine sample: 3 dietary categories (Folivory, Frugivory-Insectivory, Insectivory), unless makes with a * = Folivory, Insectivory
Primates sample: 5 dietary categories (Folivory, Frugivory, Hard-object feeder, Frugivory-Insectivory, Insectivory)
Mars m2 and/or m3 + primates sample: 6 dietary categories (Faunivory, Folivory, Frugivory, Frugivory-Insectivory, Hard-object feeder, Insectivory)

QDA combined sample classification accuracy per dietary category

QDA primates + mars m2

ariaDNE + RFI (6 diets, overall: 47.2%)

Faunivory	Folivory	Frugivory	Frugivory-Insectivory	Hard-object feeder	Insectivory
0.8000000	0.2758621	0.7000000	0.3684211	0.8888889	0.4705882

ariaDNE + RFI + lnOA (6 diets, overall: 60.7%)

Faunivory	Folivory	Frugivory	Frugivory-Insectivory	Hard-object feeder	Insectivory
0.2000000	0.5862069	0.5000000	0.7368421	0.8888889	0.5294118

ariaDNE + RFI + lnOA+ TriTaHI (6 diets, overall: 67.4%)

Faunivory	Folivory	Frugivory	Frugivory-Insectivory	Hard-object feeder	Insectivory
0.0000000	0.8275862	0.7000000	0.6315789	0.7777778	0.5882353

QDA primates + mars m3

ariaDNE + RFI (6 diets, overall: 52.9%)

Faunivory	Folivory	Frugivory	Frugivory-Insectivory	Hard-object feeder	Insectivory
0.8333333	0.3333333	0.7000000	0.4444444	0.8888889	0.5294118

ariaDNE + RFI + lnOA (6 diets, overall: 63.2%)

Faunivory	Folivory	Frugivory	Frugivory-Insectivory	Hard-object feeder	Insectivory
0.5000000	0.5555556	0.5000000	0.6666667	0.8888889	0.7058824

ariaDNE + RFI + lnOA+ TriTaHI (6 diets, overall: 70.1%)

Faunivory	Folivory	Frugivory	Frugivory-Insectivory	Hard-object feeder	Insectivory
0.3333333	0.7777778	0.6000000	0.6666667	0.8888889	0.7058824

QDA primates + mars m2 & m3

ariaDNE + RFI (*7 diets*, overall: 42.1%)

Faunivory	Folivory	Frugivory	Frugivory-Insectivory	Hard-object feeder	Insectivory	Omnivory
0.6666667	0.2333333	0.7000000	0.2631579	0.8888889	0.4705882	0.2500000

ariaDNE + RFI (6 diets, overall: 48.4%)

Faunivory	Folivory	Frugivory	Frugivory-Insectivory	Hard-object feeder	Insectivory
0.8333333	0.2666667	0.7000000	0.3684211	0.8888889	0.5294118

ariaDNE + RFI + lnOA (*7 diets*, overall: 60%)

Faunivory	Folivory	Frugivory	Frugivory-Insectivory	Hard-object feeder	Insectivory	Omnivory
0.1666667	0.6000000	0.5000000	0.7368421	0.8888889	0.6470588	0.000000

ariaDNE + RFI + lnOA (6 diets, overall: 64.8%)

Faunivory	Folivory	Frugivory	Frugivory-Insectivory	Hard-object feeder	Insectivory
0.5000000	0.6000000	0.5000000	0.7368421	0.8888889	0.6470588

ariaDNE + RFI + lnOA+ TriTaHI (6 diets, overall: 69.2%)

Faunivory	Folivory	Frugivory	Frugivory-Insectivory	Hard-object feeder	Insectivory
0.3333333	0.8333333	0.5000000	0.6315789	0.8888889	0.6470588

Table S3. Exact same sample (36 taxa) m2-only versus m3-only versus m2&m3 comparison (species averages)

Metric	Marsupial m2-only	Marsupial m3-only	Marsupial m2 & m3	Primate
RFI+ariaDNE	48.5%	57.6%	57.6%	62.7%
RFI+ariaDNE+size	45.5%	66.7%	63.6%	80.4%
RFI+ariaDNE+size+TrigTaHI	72.4%*	72.4%*	72.4%*	74.5%

Marsupial samples (m2 only, m3 only, and all): 4 different dietary categories (folivory, insectivory, frugivory-insectivory, faunivory), unless marked with a * = folivory, insectivory, faunivory)
Notes: m2 performs better in insectivore and frugivore-insectivore categories for ariaDNE and RFI than the m3 does, and better for insectivore and folivore for ariaDNE, RFI, size.

Table S4. QDA results (marsupials = training set, primates = test set) of specimen values vs. [*species averages*]. Training accuracy is calculated using the entire training sample (not leave-one-out). Training set included Faunivory, Folivory, Frugivory-Insectivory, and Insectivory categories. Accuracy of the QDA test was calculated using shared categories only, i.e., Folivory, Insectivory, and, in some cases, Frugivory-Insectivory. Results marked with an * = Frugivore-Insectivory category was excluded as the sample for this category was too small.

Train sample (train accuracy)	Train accuracy	Variables	Test sample	Test accuracy	Folivory accuracy	Frugivory-Insectivory accuracy	Insectivory accuracy
Marsupial m2	52.6% [*51.4%*]	ariaDNE + RFI	Primates	32.0% [*27.3%*]	5/32 [*3/12*]	25/53 [*5/14*]	1/12 [*1/7*]
	73.7% [*73.0%*]	ariaDNE + RFI + TriTaHI		40.2% [*36.4%*]	5/32 [*0/12*]	30/53 [*8/14*]	4/12 [*4/7*]
	**84.2% [*89.2%*]**	**ariaDNE + RFI + size**		**64.9%** [*69.7%*]	25/32 [*10/12*]	30/53 [*7/14*]	8/12 [*6/7*]
	94.7% [*94.7*]	ariaDNE + RFI + size + TriTaHI		63.9% [*63.6%*]	25/32 [*7/12*]	28/53 [*8/14*]	9/12 [*6/7*]
Marsupial m3	69.4% *[71.4%*]	ariaDNE + RFI	Primates	33.0% [*33.3%*]	13/32 [*4/12*]	10/53 [*1/14*]	9/12 [*6/7*]
	80.6% [*80.0%*]	ariaDNE + RFI + TriTaHI		25.8% [*33.3%*]	15/32 [*5/12*]	0/53 [*0/14*]	10/12 [*6/7*]
	88.9% [*91.4%*]	**ariaDNE + RFI + size**		**46.4%** [*45.5%*]	28/32 [*10/12*]	10/53 [*2/24*]	7/12 [*3/7*]
	100%* [*100%**]	ariaDNE + RFI + size + TriTaHI		82.2%* [*73.7%**]	27/32 [*8/12*]	NA	10/12 [*6/7*]
Marsupial m2 & m3	64.9% [*56.4%*]	ariaDNE + RFI	Primates	33.0% [*36.4%*]	6/32 [*4/12*]	24/53 [*4/14*]	2/12 [*4/7*]
	70.3% [*79.5%*]	ariaDNE + RFI + TriTaHI		36.1% [*36.4%*]	15/32 [*4/12*]	19/53 [*2/14*]	1/12 [*6/7*]
	90.5%	**ariaDNE + RFI + size**		**69.1%**	27/32	33/53	7/12

	[*89.7%*]			[*69.7%*]	[*10/12*]	[*7/14*]	[*6/7*]
	96.0% [*100%*]	ariaDNE + RFI + size + TriTaHI		63.9% [*48.5%*]	25/32 [*9/12*]	31/53 [*1/14*]	6/12 [*6/7*]

Table S5. QDA category-specific results with ariaDNE, RFI, size as metrics. Train sample = marsupial m2 sample (vertical categories), test sample = primate sample (horizontal categories). Bolded numbers show the results of **overlapping diets**.

	**Folivory**	Frugivory	**Frugivory-Insectivory**	Hard-object feeding	**Insectivory**	Omnivory
Faunivory	1	0	0	0	0	0
**Folivory**	**10**	7	1	9	0	0
**Frugivory-Insectivory**	1	2	**7**	0	1	1
**Insectivory**	0	0	6	0	**6**	0

Table S6. QDA category-specific results with ariaDNE, RFI, size as metrics. Train sample = marsupial m3 sample (vertical categories), test sample = primate sample (horizontal categories). Bolded numbers show the results of **overlapping diets**.

	**Folivory**	Frugivory	**Frugivory-Insectivory**	Hard-object feeding	**Insectivory**	Omnivory
Faunivory	1	0	1	0	0	0
**Folivory**	**10**	7	1	7	4	0
**Frugivory-Insectivory**	0	1	**2**	2	0	0
**Insectivory**	1	1	10	0	**3**	1

Table S7. QDA results (primates = training set, marsupials = test set) of specimen values vs. [*species averages*]. Training accuracy is calculated using the entire training sample (not leave-one-out). Training set of platyrrhine sample includes Folivory, Frugivory, Frugivory-Insectivory, Hard-object feeder, and the training set using the total primate sample included the same categories as well as the Insectivory category. Accuracy of the QDA test was calculated using shared categories only, i.e., Folivory, Frugivory, and Frugivory-Insectivory.

Train sample (train accuracy)	Train accuracy	Variables	Test sample	Test accuracy	Folivory accuracy	Frugivory accuracy	Frugivory-Insectivory accuracy	Insectivory accuracy
Platyrrhine m2	73.8%	ariaDNE + RFI	Marsupials (m3 & m3)	44.2%	14/32	0/2	5/9	NA
	80%	**ariaDNE + RFI + TriTaHI**		**48.8%**	13/32	0/2	8/9	NA
	97.9%	ariaDNE + RFI + size		32.56%	5/32	0/2	9/9	NA
	97.2%	ariaDNE + RFI + size + TriTaHI		32.56%	5/32	0/2	9/9	NA
Total primate sample	66.1% [*72.5%*]	ariaDNE + RFI	Marsupials (m3 & m3)	33.9% [*14.7%*]	16/32 [*2/18*]	0/2 [*0/1*]	1/9 [*1/5*]	5/22 [*2/10*]
	71.8% [*70.6%*]	ariaDNE + RFI + TriTaHI		30.8% [*23.5%*]	11/32 [*0/18*]	0/2 [*0/1*]	3/9 [*2/5*]	6/22 [*6/10*]
	93.7% [*92.2%*]	**ariaDNE + RFI + size**		50.8% **[*61.8%*]**	21/32 [*14/18*]	0/2 [*0/1*]	5/9 [*5/5*]	7/22 [*2/10*]
	94.8% [*94.1%*]	ariaDNE + RFI + size + TriTaHI		32.3% [*29.4%*]	14/32 [*7/18*]	0/2 [*0/1*]	5/9 [*2/5*]	2/22 [*1/10*]

Breakdown of QDA that includes **all primates** and **marsupial m2 & m3s** (last column of table 7) per dietary category:

RFI + ariaDNE (overall: 48.8%)

Faunivory	Folivory	Frugivory	Frugivory-Insectivory	Hard-object feeder	Insectivory	Omnivory
0.7272727	0.1875000	0.8205128	0.3709677	0.8750000	0.6176471	0.5000000

RFI + ariaDNE + size (overall: 64.8%)

Faunivory	Folivory	Frugivory	Frugivory-Insectivory	Hard-object feeder	Insectivory	Omnivory
0.7272727	0.7187500	0.8717949	0.8064516	0.8750000	0.7352941	0.6666667

RFI + ariaDNE + size + TriTaHI (overall: 69.2%)

Faunivory	Folivory	Frugivory	Frugivory-Insectivory	Hard-object feeder	Insectivory	Omnivory
0.9090909	0.7812500	0.8717949	0.7419355	0.8750000	0.7647059	0.3333333

RFI + ariaDNE + size + ariaDNE CV (overall: 75.8%)

Faunivory	Folivory	Frugivory	Frugivory-Insectivory	Hard-object feeder	Insectivory	Omnivory
0.7272727	0.7500000	0.8974359	0.8225806	0.9500000	0.8529412	0.5000000

RFI + ariaDNE + size + OPCR (overall: 79.1%)

Faunivory	Folivory	Frugivory	Frugivory-Insectivory	Hard-object feeder	Insectivory	Omnivory
0.8181818	0.8593750	0.8974359	0.8387097	0.9250000	0.8823529	0.3333333

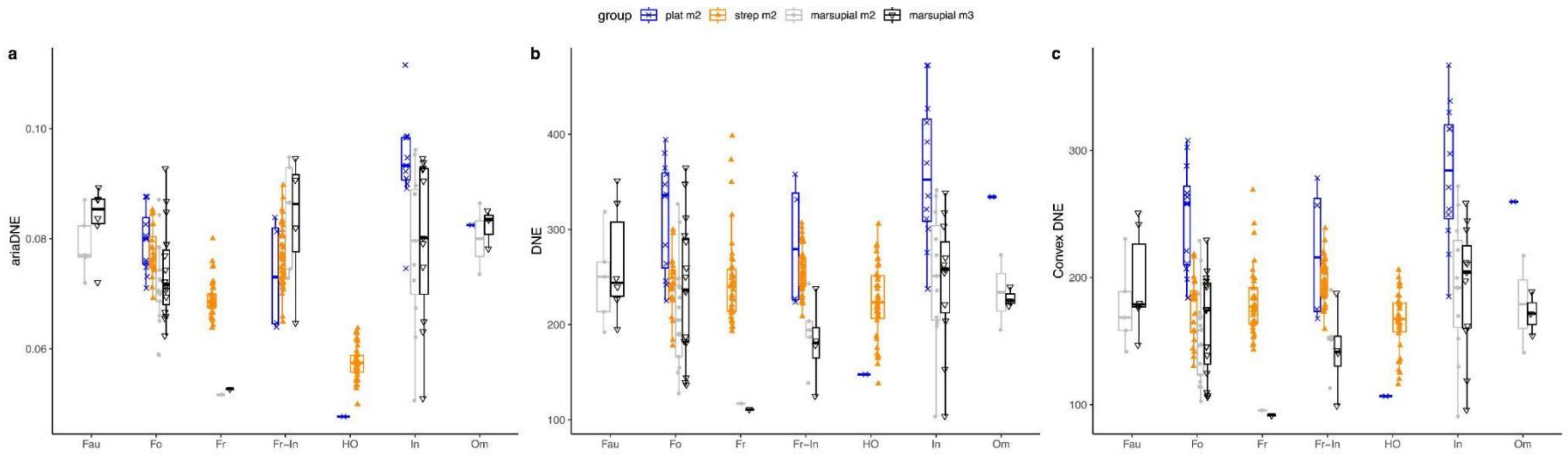

Fig. S5 Sensitivity of curvature metrics to material. Plat m2 (blue) & strep m2 (orange) are µCT scans of plastic casts. Marsupial m2 (light grey) and marsupial m3 (black) are a mix of surface and µCT scans of biological specimens.

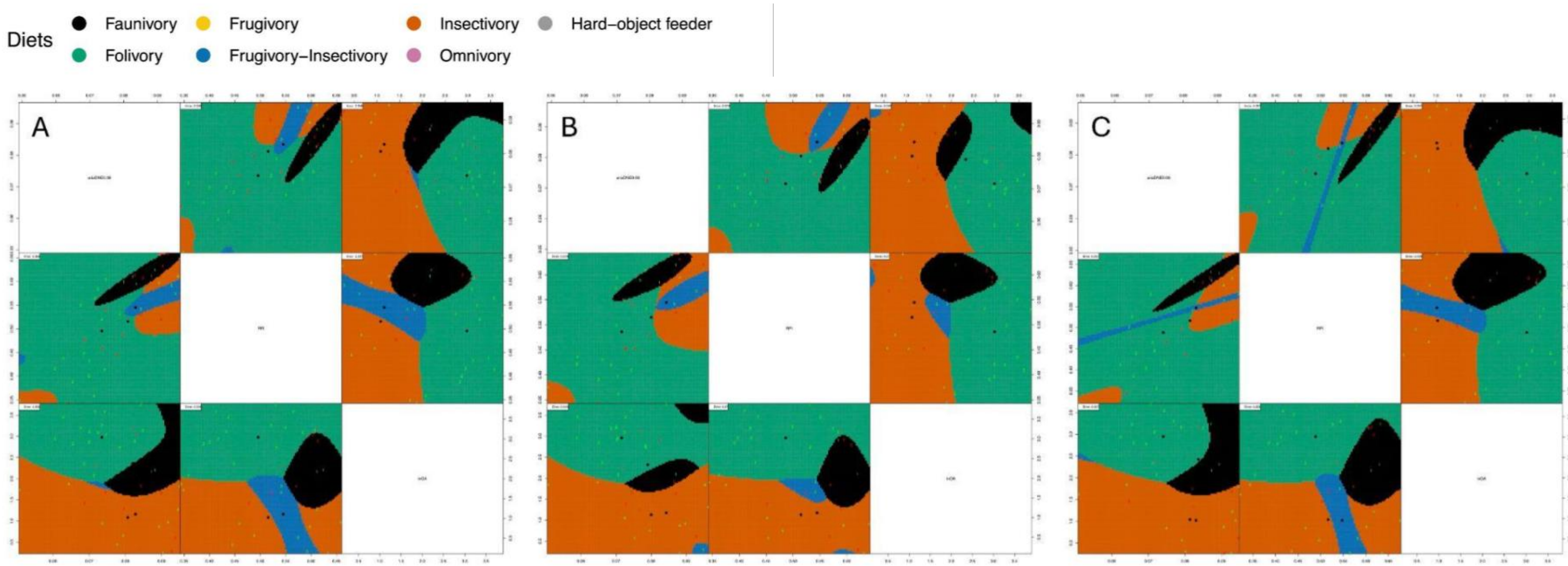

Fig. S6 A. 2D-visualisation of QDA for marsupial (m2 & m3) species averages. B. marsupial m2-only. C. marsupial m3-only.
1 = faunivory, 2 = folivory, 3 = frugivory-insectivory, 4 = insectivory. Green number = correct, red number = incorrect in the training sample.

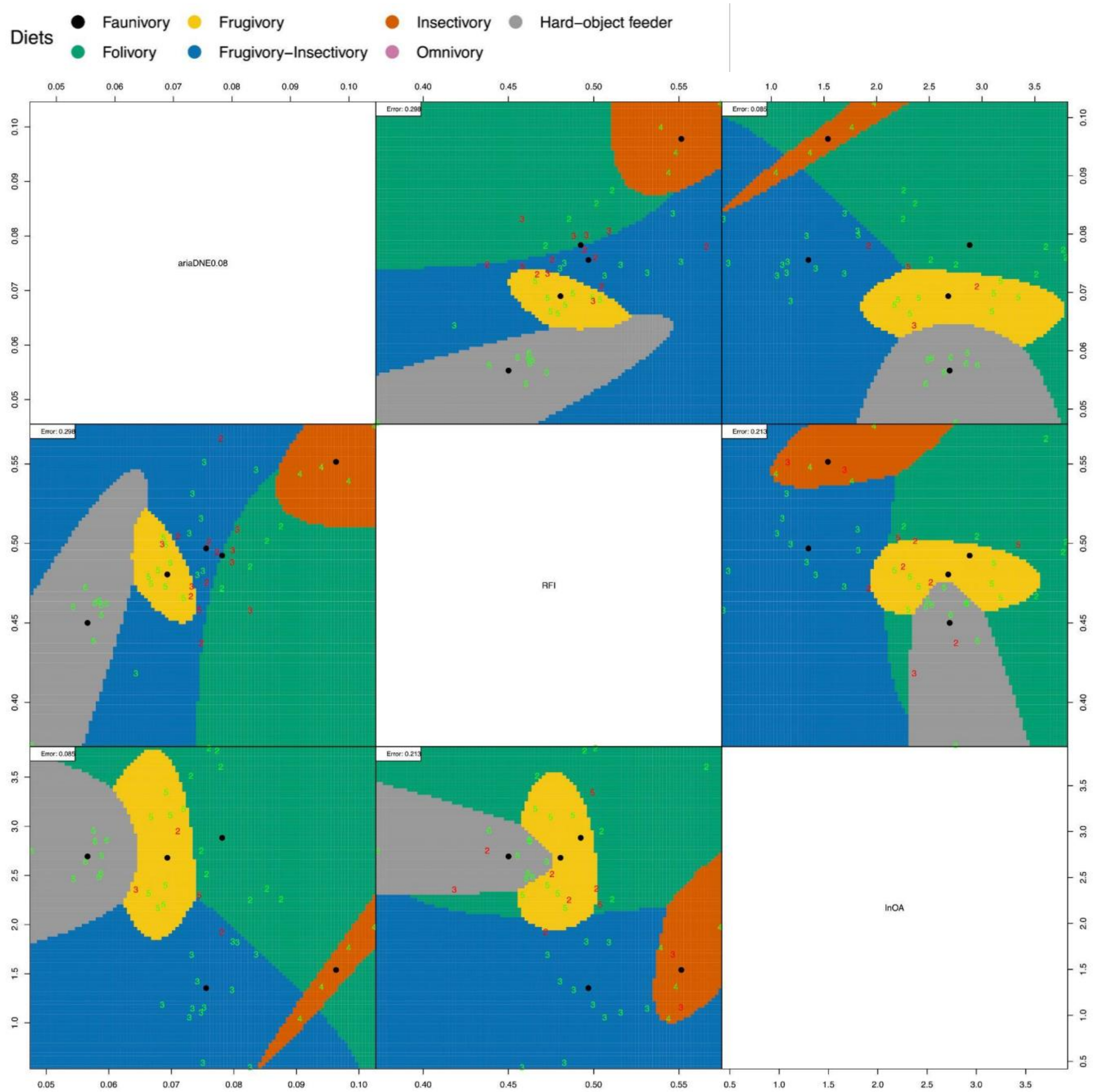

Fig. S7 2D-visualisation of QDA for <u>primate-only</u> species averages. 2 = folivory, 3 = frugivory-insectivory, 4 = insectivory, 5 = frugivory. Green number = correct, red number = incorrect in the training sample.

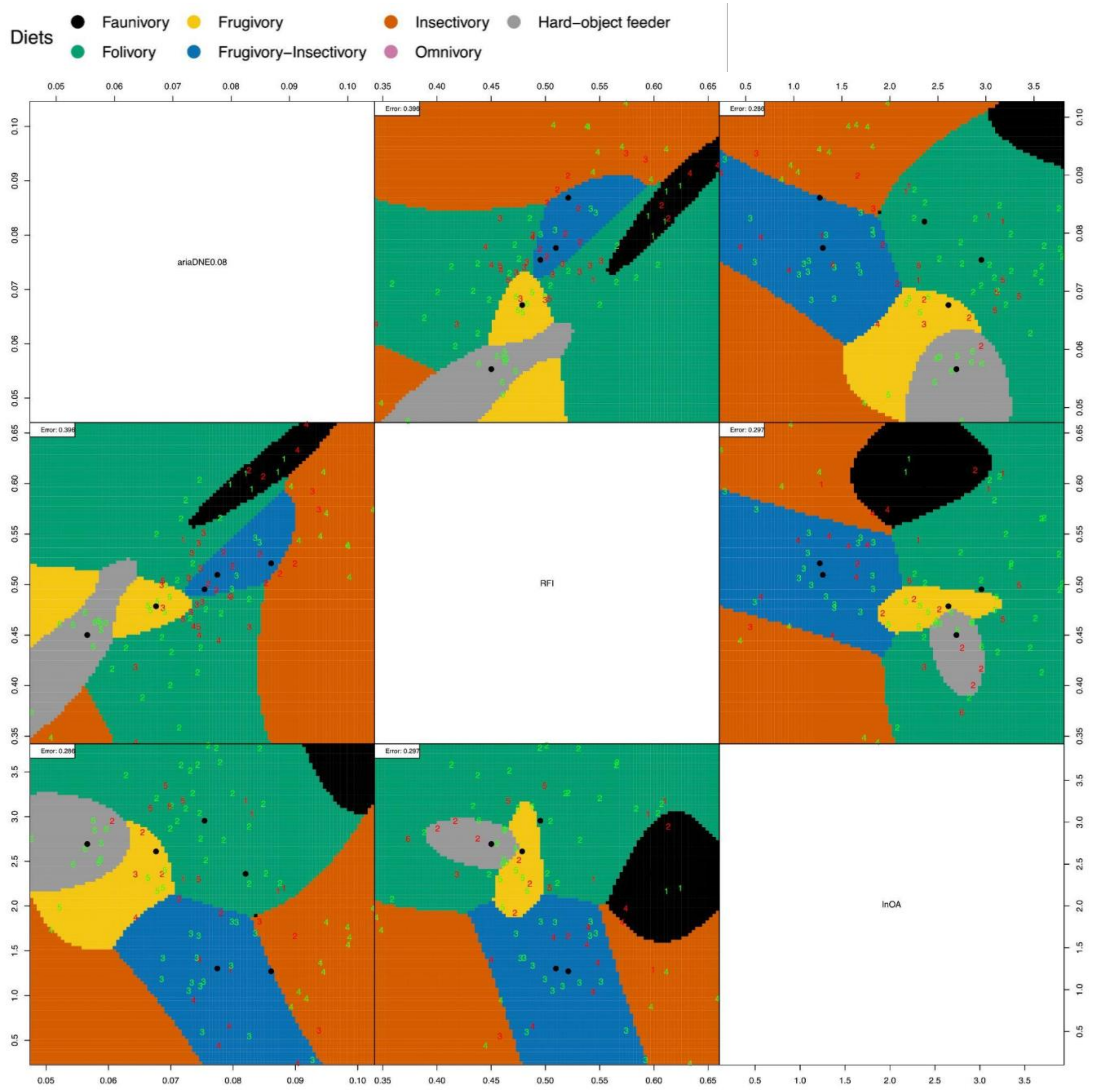

Fig. S8 2D-visualisation of QDA for total sample of marsupial (m2 & m3) and primate species averages. 1 = faunivory, 2 = folivory, 3 = frugivory-insectivory, 4 = insectivory, 5 = frugivory, 6 = hard-object feeder. Green number = correct, red number = incorrect in training sample.

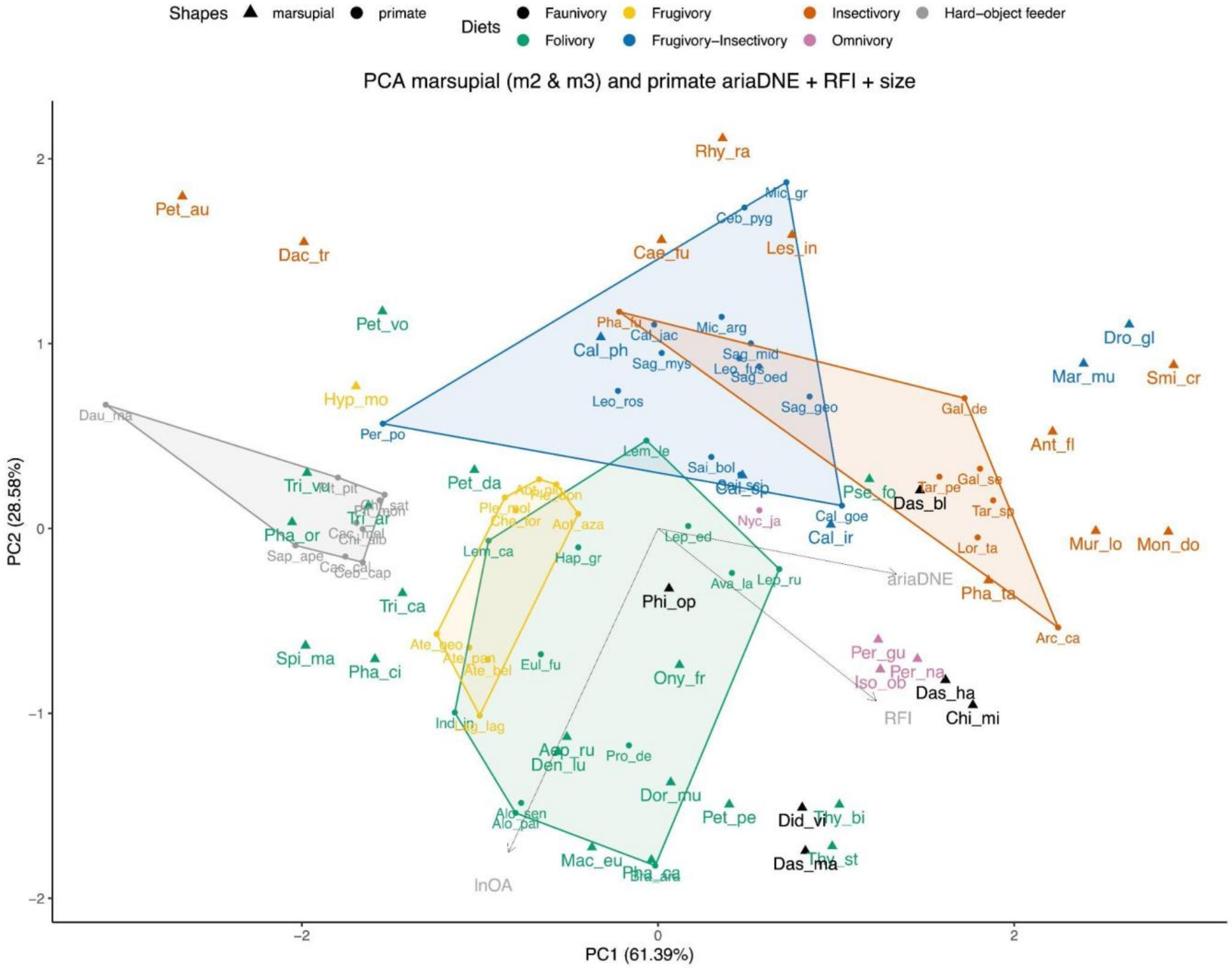

Fig. S9 Principal Component Analysis of ariaDNE, RFI, and lnOA plot showing PC1 and PC2 scores for total marsupial (m2 & m3) and primate species averaged data, capturing 89.97% of the variation. Note that the PC loading arrows are not to scale. Convex hulls are primate-only. Marsupial taxon abbreviations can be found in Table 3.

Table S8. MorphoSource files information and funding acknowledgements.

File	resolution	data uploaded by	Funding acknowledgement listed on MorphoSource	doi and ARK
Aepyprymnus_rufescens_AMNH_22788	0.057922	Arianna Harrington	NSF BCS 1825129 (to D.M. Boyer and A.R. Harrington)	doi:10.17602/M2/M76018
Antechinus_flavipes_SAMA_M27013	0.026	Jacob van Zoelen	CABAH, Weisbecker, V., Fusco, D., Ingleby, S., Lambrides, A.B.J., Manne, T., Maguire, K., O'Connor, S., Peachey, T.J., Samper Carro, S.C., Stemmer, D., Ristevski, J., van Zoelen, J.D., Viacava, P., Yates, A.M., and Mein, E. 2025 Ozboneviz: an Australian precedent in FAIR 3D imagery and extended biodiversity collections, BioScience, p. biaf064, https://doi.org/10.1093/biosci/biaf064	ark:/87602/m4/508065
Caenolestes_fuliginosus_KU124015	0.014,0.014, 0.03	Jessie Maisano	Ted Macrini provided access to these data, with data collection funded by NSF DEB-0309369 and data upload to MorphoSource funded by DBI-1902242. The files were downloaded from www.MorphoSource.org, Duke University.	ark:/87602/m4/M116130
Caluromys_philander_AMNH95526	0.03711,0.037110, 0.079950	Jessie Maisano	Ted Macrini provided access to these data, with data collection funded by NSF DEB-0309369 and data upload to MorphoSource funded by DBI-1902242. The files were downloaded from www.MorphoSource.org, Duke University.	ark:/87602/m4/M116113
Caluromys_sp_DU_EA_162_m2	0.042338	Arianna Harrington	NSF BCS 1552848 (to D M Boyer), NSF DBI 1458192 (to G F Gunnell)	doi:10.17602/M2/M58092
Caluromysiops_irrupta_FMNH60698	0.089676	Roger Benson	Roger Benson provided access to these data originally appearing in Marin-Serra A, Benson RBJ. 2020. Developmental constraints do not influence long-term phenotypic evolution of marsupial forelimbs as revealed by interspecific disparity and integration patterns. American Naturalist 195(3) https://doi.org/10.1086/707194, the collection of which was funded by the European Research Council (ERC) starting grant TEMPO (ERC-2015-STG-677774) to Roger Benson. The files were downloaded from www.MorphoSource.org, Duke University.	ark:/87602/m4/M82280
Chironectes_minimus_AMNH129701	0.03223,0.032230, 0.035120	Jessie Maisano	Jeri Rodgers provided access to these data, with data upload to MorphoSource funded by DBI-1902242. The files were downloaded from www.MorphoSource.org, Duke University.	ark:/87602/m4/M114558
Dactylopsila_trivirgata_AMNH101984	0.017284	Mary Silcox	Mary T. Silcox provided access to these data NSERC Discovery Grant to Mary T. Silcox. The files were downloaded from www.MorphoSource.org, Duke University.	ark:/87602/m4/M75882
Dactylopsila_trivirgata_AMNH101985	0.016863	Mary Silcox	Mary T. Silcox provided access to these data NSERC Discovery Grant to Mary T. Silcox. The files were downloaded from www.MorphoSource.org, Duke University.	ark:/87602/m4/M75886
Dasycercus_blythi_SAMA_M3635	0.036	Jacob van Zoelen	CABAH, Weisbecker, V., Fusco, D., Ingleby, S., Lambrides, A.B.J., Manne, T., Maguire, K., O'Connor, S., Peachey, T.J., Samper Carro, S.C., Stemmer, D., Ristevski, J., van Zoelen, J.D., Viacava, P., Yates, A.M., and Mein, E. 2025 Ozboneviz: an Australian precedent in FAIR 3D imagery and extended biodiversity collections, BioScience, p. biaf064, https://doi.org/10.1093/biosci/biaf064	ark:/87602/m4/508066
Dasyurus_hallucatus_SAMA_M15386	0.054	Jacob van Zoelen	CABAH, Weisbecker, V., Fusco, D., Ingleby, S., Lambrides, A.B.J., Manne, T., Maguire, K., O'Connor, S., Peachey, T.J., Samper Carro, S.C., Stemmer, D., Ristevski, J., van Zoelen, J.D., Viacava, P., Yates, A.M., and Mein, E. 2025 Ozboneviz: an Australian precedent in FAIR 3D imagery and extended biodiversity collections, BioScience, p. biaf064, https://doi.org/10.1093/biosci/biaf064	ark:/87602/m4/508067
Dasyurus_maculatus_SAMA_M27408	mandible = 4867150 polygons	Erin Mein	ARC Centre of Excellence for Australian Biodiversity and Heritage, Weisbecker, V., Fusco, D., Ingleby, S., Lambrides, A.B.J., Manne, T., Maguire, K., O'Connor, S., Peachey, T.J., Samper Carro, S.C., Stemmer, D., Ristevski, J., van Zoelen, J.D., Viacava, P., Yates, A.M., and Mein, E. 2025 Ozboneviz: an Australian precedent in FAIR 3D imagery and extended biodiversity collections, BioScience, p. biaf064, https://doi.org/10.1093/biosci/biaf064	ark:/87602/m4/537548
Dendrolagus_lumholtzi_AMNH65254	0.068800,0.068800,0.160200	Jessie Maisano	Ted Macrini provided access to these data, with data collection funded by NSF DEB-0309369 and data upload to MorphoSource funded by DBI-1902242. The files were downloaded from www.MorphoSource.org, Duke University.	ark:/87602/m4/M116135
Didelphis_virginiana_TMM_M_2517	0.059600,0.059600,0.132000	Jessie Maisano	Ted Macrini provided access to these data, with data collection funded by NSF DEB-0309369 and data upload to MorphoSource funded by DBI-1902242. The files were downloaded from www.MorphoSource.org, Duke University.	ark:/87602/m4/M116136

File	resolution	data uploaded by	Funding acknowledgement listed on MorphoSource	doi and ARK
Dorcopsis_muelleri_SAM_M13754	mandible = 3337923	Jorgo Ritsevski	CABAH, Weisbecker, V., Fusco, D., Ingleby, S., Lambrides, A.B.J., Manne, T., Maguire, K., O'Connor, S., Peachey, T.J., Samper Carro, S.C., Stemmer, D., Ristevski, J., van Zoelen, J.D., Viacava, P., Yates, A.M., and Mein, E. 2025 Ozboneviz: an Australian precedent in FAIR 3D imagery and extended biodiversity collections, BioScience, p. biaf064, https://doi.org/10.1093/biosci/biaf064	10.17602/M2/M592786
Dromiciops_gliroides_FMNH_127463	0.016100,0.016100,0.039500	Jessie Maisano	Ted Macrini provided access to these data, with data collection funded by NSF DEB-0309369 and data upload to MorphoSource funded by DBI-1902242. The files were downloaded from www.MorphoSource.org, Duke University.	ark:/87602/m4/M116137
Hypsiprymnodon_moschatus_AMNH184580	0.037210,0.037210,0.079920	Barbara Sulbaran	Ted Macrini provided access to these data with data collection funded by NSF DEB-0309369 and data upload to MorphoSource funded by DBI-1902242. The files were downloaded from www.MorphoSource.org, Duke University.	ark:/87602/m4/M117097
Isoodon_obesulus_SAMA_M25975	0.056	Diana Fusco	CABAH, Weisbecker, V., Fusco, D., Ingleby, S., Lambrides, A.B.J., Manne, T., Maguire, K., O'Connor, S., Peachey, T.J., Samper Carro, S.C., Stemmer, D., Ristevski, J., van Zoelen, J.D., Viacava, P., Yates, A.M., and Mein, E. 2025 Ozboneviz: an Australian precedent in FAIR 3D imagery and extended biodiversity collections, BioScience, p. biaf064, https://doi.org/10.1093/biosci/biaf064	ark:/87602/m4/536302
Lestoros_inca_MVZ116049	0.017957	Arianna Harrington	NSF BCS 1552848 (to D M Boyer), NSF DBI 1458192 (to G F Gunnell)	doi:10.17602/M2/M57778
Macropus_eugenii_TMM_M1047	0.0623	Barbara Sulbaran	Ted Macrini provided access to these data, with data collection funded by NSF DEB-0309369 and data upload to MorphoSource funded by DBI-1902242. The files were downloaded from www.MorphoSource.org, Duke University.	10.17602/M2/M167398
Marmosa_murina_NHMUK_1881	0.041269	Roger Benson	Roger Benson provided access to these data originally appearing in Marin-Serra A, Benson RBJ. 2020. Developmental constraints do not influence long-term phenotypic evolution of marsupial forelimbs as revealed by interspecific disparity and integration patterns. American Naturalist 195(3) https://doi.org/10.1086/707194, the collection of which was funded by the European Research Council (ERC) starting grant TEMPO (ERC-2015-STG-677774) to Roger Benson. The files were downloaded from www.MorphoSource.org, Duke University.	ark:/87602/m4/M68271
Monodelphis_domestica_TMMM_7599	0.045000,0.045000,0.090000	Jessie Maisano	Tim Rowe and Ted Macrini provided access to these data, with data upload to MorphoSource funded by DBI-1902242. The files were downloaded from www.MorphoSource.org, Duke University.	10.17602/M2/M168948
Murexia_longicaudata_SAMA_M2816	0.35	Diana Fusco	CABAH, Weisbecker, V., Fusco, D., Ingleby, S., Lambrides, A.B.J., Manne, T., Maguire, K., O'Connor, S., Peachey, T.J., Samper Carro, S.C., Stemmer, D., Ristevski, J., van Zoelen, J.D., Viacava, P., Yates, A.M., and Mein, E. 2025 Ozboneviz: an Australian precedent in FAIR 3D imagery and extended biodiversity collections, BioScience, p. biaf064, https://doi.org/10.1093/biosci/biaf064	ark:/87602/m4/557696
Onychogale_frenta_UMZCa12_59_3	0.098246,0.098246,0.196491	Roger Benson	Roger Benson provided access to these data originally appearing in Marin-Serra A, Benson RBJ. 2020. Developmental constraints do not influence long-term phenotypic evolution of marsupial forelimbs as revealed by interspecific disparity and integration patterns. American Naturalist 195(3) https://doi.org/10.1086/707194, the collection of which was funded by the European Research Council (ERC) starting grant TEMPO (ERC-2015-STG-677774) to Roger Benson. The files were downloaded from www.MorphoSource.org, Duke University.	ark:/87602/m4/M82448
Perameles_gunnii_NTMU7600	mandible = 645312 polygons	Diana Fusco	CABAH, Weisbecker, V., Fusco, D., Ingleby, S., Lambrides, A.B.J., Manne, T., Maguire, K., O'Connor, S., Peachey, T.J., Samper Carro, S.C., Stemmer, D., Ristevski, J., van Zoelen, J.D., Viacava, P., Yates, A.M., and Mein, E. 2025 Ozboneviz: an Australian precedent in FAIR 3D imagery and extended biodiversity collections, BioScience, p. biaf064, https://doi.org/10.1093/biosci/biaf064	ark:/87602/m4/538502
Perameles_nasuta_MAGNT_U7608	mandible = 69827 polygons	Diana Fusco	CABAH, Weisbecker, V., Fusco, D., Ingleby, S., Lambrides, A.B.J., Manne, T., Maguire, K., O'Connor, S., Peachey, T.J., Samper Carro, S.C., Stemmer, D., Ristevski, J., van Zoelen, J.D., Viacava, P., Yates, A.M., and Mein, E. 2025 Ozboneviz: an Australian precedent in FAIR 3D imagery and extended biodiversity collections, BioScience, p. biaf064, https://doi.org/10.1093/biosci/biaf064	ark:/87602/m4/546239
Petauroides_volans_QM_J4643	0.036	Jessica Ivory-Church	Australian Research Council Discovery Early Career Award DE120102034	private file of Vera Weisbecker
Petaurus_australis_QM_J8598	0.036	Jessica Ivory-Church	Australian Research Council Discovery Early Career Award DE120102035	private file of Vera Weisbecker

File	resolution	data uploaded by	Funding acknowledgement listed on MorphoSource	doi and ARK
Petrogale_penicillata_FMNH64435	0.068177,0.068177,0.136354	Roger Benson	Roger Benson provided access to these data originally appearing in Marin-Serra A, Benson RBJ. 2020. Developmental constraints do not influence long-term phenotypic evolution of marsupial forelimbs as revealed by interspecific disparity and integration patterns. American Naturalist 195(3) https://doi.org/10.1086/707194, the collection of which was funded by the European Research Council (ERC) starting grant TEMPO (ERC-2015-STG-677774) to Roger Benson. The files were downloaded from www.MorphoSource.org, Duke University.	ark:/87602/m4/M82308
Petropseudes_dahli_AMNH183391	0.044920,0.044920,0.048660	Jessie Maisano	Jeri Rodgers provided access to these data, with data upload to MorphoSource funded by DBI-1902242. The files were downloaded from www.MorphoSource.org, Duke University.	ark:/87602/m4/M114566
Phalanger_carmelitae_SAMA_M2901	0.043	Diana Fusco	CABAH, Weisbecker, V., Fusco, D., Ingleby, S., Lambrides, A.B.J., Manne, T., Maguire, K., O'Connor, S., Peachey, T.J., Samper Carro, S.C., Stemmer, D., Ristevski, J., van Zoelen, J.D., Viacava, P., Yates, A.M., and Mein, E. 2025 Ozboneviz: an Australian precedent in FAIR 3D imagery and extended biodiversity collections, BioScience, p. biaf064, https://doi.org/10.1093/biosci/biaf064	ark:/87602/m4/535483
Phalanger_orientalis_AMNH157211	0.054000,0.054000,0.121	Barbara Sulbaran	Ted Macrini provided access to these data , with data collection funded by NSF DEB-0309369 and data upload to MorphoSource funded by DBI-1902242. The files were downloaded from www.MorphoSource.org, Duke University.	ark:/87602/m4/M167380
Phascogale_tapoatafa_SAMA_M3824	mandible = 11072904 polygons	Diana Fusco	CABAH, Weisbecker, V., Fusco, D., Ingleby, S., Lambrides, A.B.J., Manne, T., Maguire, K., O'Connor, S., Peachey, T.J., Samper Carro, S.C., Stemmer, D., Ristevski, J., van Zoelen, J.D., Viacava, P., Yates, A.M., and Mein, E. 2025 Ozboneviz: an Australian precedent in FAIR 3D imagery and extended biodiversity collections, BioScience, p. biaf064, https://doi.org/10.1093/biosci/biaf064	ark:/87602/m4/535831
Phascolarctos_cinereus_AMNH65608	0.08338	Arianna Harrington	NSF BCS 1825129 (to D.M. Boyer and A.R. Harrington)	doi:10.17602/M2/M99914
Philander_opossum_QM_J3461	0.053	Vera Weisbecker	Australian Research Council DE120102034; DP170103227	private file of Vera Weisbecker
Pseudochirulus_forbesi_AMNH104136	0.03223,0.032230,0.035470	Jessie Maisano	Jeri Rodgers provided access to these data, with data upload to MorphoSource funded by DBI-1902242. The files were downloaded from www.MorphoSource.org, Duke University.	ark:/87602/m4/M114568
Rhyncholestes_raphanurus_MVZ163773	0.018392	Arianna Harrington	NSF BCS 1552848 (to D M Boyer), NSF DBI 1458192 (to G F Gunnell)	doi:10.17602/M2/M57244
Smithopsis_crassicaudata_SAMA_M12809	0.023	Jacob van Zoelen	CABAH, Weisbecker, V., Fusco, D., Ingleby, S., Lambrides, A.B.J., Manne, T., Maguire, K., O'Connor, S., Peachey, T.J., Samper Carro, S.C., Stemmer, D., Ristevski, J., van Zoelen, J.D., Viacava, P., Yates, A.M., and Mein, E. 2025 Ozboneviz: an Australian precedent in FAIR 3D imagery and extended biodiversity collections, BioScience, p. biaf064, https://doi.org/10.1093/biosci/biaf064	ark:/87602/m4/508327
Spilocuscus_maculatus_FMNH31752	0.075973,0.075973,0.151946	Roger Benson	Roger Benson provided access to these data, the collection of which was funded by the European Research Council (ERC) starting grant TEMPO (ERC-2015-STG-677774) to Roger Benson. The files were downloaded from www.MorphoSource.org, Duke University.	ark:/87602/m4/M164405
Thylogale_billardierii_SAMAM2868	mandible = 1215244 polygons	Jacob van Zoelen	CABAH, Weisbecker, V., Fusco, D., Ingleby, S., Lambrides, A.B.J., Manne, T., Maguire, K., O'Connor, S., Peachey, T.J., Samper Carro, S.C., Stemmer, D., Ristevski, J., van Zoelen, J.D., Viacava, P., Yates, A.M., and Mein, E. 2025 Ozboneviz: an Australian precedent in FAIR 3D imagery and extended biodiversity collections, BioScience, p. biaf064, https://doi.org/10.1093/biosci/biaf064	ark:/87602/m4/568545
Thylogale_stigmatica_MAGNT_U8203	mandible = 2039374	Diana Fusco	CABAH, Weisbecker, V., Fusco, D., Ingleby, S., Lambrides, A.B.J., Manne, T., Maguire, K., O'Connor, S., Peachey, T.J., Samper Carro, S.C., Stemmer, D., Ristevski, J., van Zoelen, J.D., Viacava, P., Yates, A.M., and Mein, E. 2025 Ozboneviz: an Australian precedent in FAIR 3D imagery and extended biodiversity collections, BioScience, p. biaf064, https://doi.org/10.1093/biosci/biaf064	ark:/87602/m4/538617
Trichosurus_arnhemensis_MAGNT_U5993	mandible = 746401 polygons	Diana Fusco	CABAH, Weisbecker, V., Fusco, D., Ingleby, S., Lambrides, A.B.J., Manne, T., Maguire, K., O'Connor, S., Peachey, T.J., Samper Carro, S.C., Stemmer, D., Ristevski, J., van Zoelen, J.D., Viacava, P., Yates, A.M., and Mein, E. 2025 Ozboneviz: an Australian precedent in FAIR 3D imagery and extended biodiversity collections, BioScience, p. biaf064, https://doi.org/10.1093/biosci/biaf064	ark:/87602/m4/544616
Trichosurus_caninus_MAGNT_U7	mandible =	Diana Fusco	CABAH, Weisbecker, V., Fusco, D., Ingleby, S., Lambrides, A.B.J., Manne, T., Maguire, K., O'Connor, S.,	10.17602/M2/M546257

File	resolution	data uploaded by	Funding acknowledgement listed on MorphoSource	doi and ARK
946	1809823 polygons		Peachey, T.J., Samper Carro, S.C., Stemmer, D., Ristevski, J., van Zoelen, J.D., Viacava, P., Yates, A.M., and Mein, E. 2025 Ozboneviz: an Australian precedent in FAIR 3D imagery and extended biodiversity collections, BioScience, p. biaf064, https://doi.org/10.1093/biosci/biaf064	
Trichosurus_vulpecula_TMM849	0.053000,0.053000,0.116000	Barbara Sulbaran	Ted Macrini provided access to these data, with data collection funded by NSF DEB-0309369 and data upload to MorphoSource funded by DBI-1902242. The files were downloaded from www.MorphoSource.org, Duke University.	ark:/87602/m4/M167392

Martín-Serra A., Benson R.B. 2020 Developmental constraints do not influence long-term phenotypic evolution of marsupial forelimbs as revealed by interspecific disparity and integration patterns. The American Naturalist 195(3):547-60. https://doi.org/10.1086/707194

Weisbecker, V., Fusco, D., Ingleby, S., Lambrides, A.B.J., Manne, T., Maguire, K., O'Connor, S., Peachey, T.J., Samper Carro, S.C., Stemmer, D., Ristevski, J., van Zoelen, J.D., Viacava, P., Yates, A.M., and Mein, E. 2025 Ozboneviz: an Australian precedent in FAIR 3D imagery and extended biodiversity collections, BioScience, p. biaf064, https://doi.org/10.1093/biosci/biaf064

Dietary category justifications

From de Vries et al. (2024 table 3):

Exudate feeders: Fruit = 0.28, Animal matter = 0.21, Exudates = 0.52

Hard-object feeders (‘seed eaters’ in original source): Seeds >0.32, Fruit = 0.19-0.47, Leaves <0.1, Animal matter < 0.1

Folivore: Leaves >0.5, Fruit 0.4-0.46

Frugivore: Fruit > 0.58, Leaves 0.12-0.28, Animal matter <0.13

Frugivore-insectivore: Fruit >0.56, Leaves <0.06, Animal matter >0.13

Additional categories:

Insectivore: >50% insects, less fruit consumption than in the frugivore-insectivore category

Faunivore: regular consumption of vertebrate prey

Omnivore: animals, fruit, and non-reproductive parts of plants (e.g. leaves, roots) all form major parts of the diet

Primates

Avahi laniger: folivore (Harcourt 1991; Faulkner and Lehman 2006).

Galagoides demidoff: insectivore - diet is primarily insects: 70% insects, 19% fruit, 10% gums, and a very small amount of leaves and buds (Charles-Dominique 1977; Hladik 1979).

Galago senegalensis: insectivore - primary diet is insects and acacia gum, so if exudates are excluded, then, it’s insectivore (Harcourt 1986).

Lemur catta: folivore - diet can be >50% leaves at certain times of the year (Simmen et al. 2003; Gould et al. 2011:2, 3).

Indri indri: folivore (Britt et al. 2002; Powzyk and Mowry 2003, 2006; Randrianarison et al. 2022).

Lepilemur edwardsi: folivore (Thalmann 2001).

Lepilemur leucopus: folivore (Dröscher and Kappeler 2014).

Lepilemur ruficaudatus: folivore (Ganzhorn et al. 2004).

Microcebus griseorufus: frugivore-insectivore (Crowley et al. 2014:2) - eats considerable amounts of exudates, but if excluded then diet is predominately fruit and insects.

Phaner furcifer: insectivore - diet consists nearly entirely of tree exudates and insect secretions (Petter et al. 1971) and its exudativorous diet is complemented with insects (Charles-Dominique and Petter 1980; only about 10%, Hladik et al. 1980).

Propithecus deckenii: folivore (55%, Fabrice et al. 2024).

Tarsius pelengensis: insectivore (Syahrullah et al. 2023).

Hapalemur griseus: folivore, >80% bamboo or other leafy materials (Grassi 2006).

Arctocebus calabarensis: insectivore (Charles-Dominique 1977).

Daubentonia madagascariensis: hard-object feeder based on >25% of extremely hard shelled *Canarium* seeds in their diet (Sterling 1994; Randimbiharinirina et al. 2018), see main text for full justification

Eulemur fulvus: folivore (Sussman 1977; Ganzhorn 1986; Overdorff 1993).

Loris tardigradus: insectivore (Nekaris and Jayewardene 2003).

Nycticebus javanicus: exudativorous (gums and nectar) according to Cabana et al. (2017, figure 1), but when exudates are excluded, its diet consists mostly of insects and leaves, with a lower contribution of flowers and fruits (based on % time feeding), and we therefore group it as an omnivore.

Perodicticus potto: frugivorous with a considerable contribution of gums and insects to its diet (Nekaris and Bearder 2007). As we exclude the exudativorous component, we classify it as a frugivore-insectivore.

Marsupials

Caenolestes fuliginosus: highly insectivorous based on gut contents, with lepidopteran larvae,

centipedes, unidentified arachnids making up 75% of gut contents of eight specimens, both in volume and in frequency of being present in each specimen (Barkley and Whitaker 1984).
Lestoros inca: insectivore very little published on its behaviour and diet. We classified this as an insectivore following (Siciliano Martina 2013), based on general Canaestolidae behaviour.
Rhyncholestes raphanurus: insectivore as it mainly consumed invertebrates such as arthropods and annelids (54.7%), with its secondary dietary component being made of by plant material and fungi (39%, Meserve et al. 1988).
Caluromys philander: frugivore-insectivore. Classified as frugivore by Julien-Laferrière (1999), with the majority of its diet being fruits. But an estimate of 25% of insects in its diet is reported (Atramentowicz 1982; Julien-Laferrière 1999), therefore being an Frugivore-insectivore in our dietary scheme.
Caluromys sp: frugivore-insectivore, following our classification of *Caluromys philander.*
Caluromysiops irrupta: frugivore-insectivore. Frugivore-omnivore according to Robinson and Redford (1986), which is defined as >50% fruits, remainder mostly invertebrates and vertebrates.
Chironectes minimus: faunivore, its diet consists mainly of crustaceans (Medellín 1991) and fishes (Mondolfi and Padilla 1958; Galliez et al. 2009).
Didelphis virginiana: faunivore. Its diet is described as being highly opportunistic, with the bulk being made up of animal foods, mostly vertebrate prey and insects (McManus 1974).
Marmosa murina: frugivore-insectivore. A reported 80% of studied gut contents had arthropods present, compared to 100% of gut specimens containing fruit (Parreira Claro and Hannibal 2022).
Monodelphis domestica: insectivore. Wild populations focus on predating on invertebrates, whereas captive members also accept vertebrates into their diet in the laboratory (Streilein 1982).
Philander opossum: faunivore. They feed on small mammals, birds, eggs, carrion, and fruit (Hunsaker 2012).
Dactylopsila trivirgata: insectivore, mostly feeding on invertebrates (Rawlins and Handasyde 2002).
Petaurus australis: insectivore. The bulk of its diet is exudates, but this is supplemented by a variety of arthropods (Smith and Russell 1982; Harcourt 1986; Schülke 2003 fig. 2).
Aepyprymnus rufescens: grasses, herbaceous plants, roots, and fungi (Baker and Gynther 2023).
Dendrolagus lumholtzi: folivore as its diet is mainly rainforest foliage (Martin 2005).
Macropus eugenii: folivore as its diet mainly consists of grasses and foliage (Williamson 1986).
Onychogalea frenata: folivore, as its diet consists mainly of herbaceous plants (Dawson et al. 1992); 40-50% graze and the rest browse (Tierney 1985).
Petrogale penicillata: folivore, as its diet consisted of 10–40% grass, 30–50% browse, 12–45% forbs and minor quantities of orchid/lilies and sedges (Tuft et al. 2011).
Dromiciops gliroides: insectivore-frugivore
Phascolarctos cinereus: specialised folivore of *Eucalyptus* (Marsh et al. 2021; Eisenhofer et al. 2023)
Petauroides volans: folivore, with its diet nearly exclusively consisting of young leaves and flower buds of a few *Eucalyptus* species (Harris and Maloney 2010).
Petropseudes dahli: folivore as the primary component of its diet is leaves, although it occasionally consumes flowers and fruit as well (Runcie 2002).
Pseudochirulus forbesi: folivore (Hume et al. 1993; Stephens et al. 2006)
Phalanger orientalis: folivore, with their diet consisting of leaves, fruit, and bark (Farida 2022).
Spilocuscus maculatus: folivore with foliage, fruits, and shoots being the most common parts of its diet. It is described as being principally folivorous and partially frugivorous by Heinsohn (2002), or as feeding primarily on fruits and leaves (Latinis 1996). However, according to Saragih et al. (2010): "Approximately 64.4% of cuscus diets is the combination between pulp of fruit and epidermis of fruit, while 21.1% is shoot". As it is inconclusive whether the largest component of the diet of *S. maculatus* is fruits or leaves, we assign this taxon to being a folivore, in which the proportions of leaves to fruits are closest to 50:50 (Leaves >0.5, Fruit 0.4-0.46, see above), in contrast to our definition of frugivores for which fruits are at least double as abundant in the diet as leaves are (Fruit > 0.58, Leaves < 0.28).

Trichosurus vulpecula: folivore. Highly variable diet, but leaves comprising the majority of its diet (Wilson and Mittermeier 2015).

REFERENCES

Atramentowicz M (1982) Influence du milieu sur l'activité locomotrice et la reproduction de *Caluromys philander* (L.). Rev écol 36:373–395

Baker AM, Gynther IC (eds) (2023) Strahan's mammals of Australia. Bloomsbury Publishing PLC, London

Barkley LJ, Whitaker J (1984) Confirmation of *Caenolestes* in Peru with information on diet. J Mammal 65:328–330. https://doi.org/10.2307/1381173

Britt A, Randriamandratonirina NJ, Glasscock KD, Iambana BR (2002) Diet and feeding behaviour of *Indri indri* in a low-altitude rain forest. Folia Primatol 73:225–239. https://doi.org/10.1159/000067455

Cabana F, Dierenfeld E, Wirdateti W, Donati G, Nekaris KAI (2017) The seasonal feeding ecology of the javan slow loris (*Nycticebus javanicus*). Am J Phys Anthropol 162:768–781. https://doi.org/10.1002/ajpa.23168

Charles-Dominique P (1977) Ecology and behaviour of nocturnal primates: Prosimians of equatorial west Africa. Duckworth, London

Charles-Dominique P, Petter J (1980) Ecology and Social Life of Phaner furcifer. In: Charles-Dominique P et al. (eds) Nocturnal Malagasy primates. Academic Press New York, New York, pp 75–95. https://doi.org/10.1016/B978-0-12-169350-3.50008-3

Crowley BE, Rasoazanabary E, Godfrey LR (2014) Stable isotopes complement focal individual observations and confirm dietary variability in reddish-gray mouse lemurs (*Microcebus griseorufus*) from southwestern Madagascar: Assessing mouse lemur diet with stable isotopes. Am J Phys Anthropol 155:77–90. https://doi.org/10.1002/ajpa.22555

Dawson T, Tierney P, Ellis B (1992) The diet of the Bridled nailtail wallaby (*Onychogalea fraenata*). II Overlap in dietary niche breadth and plant preferences with the black-striped wallaby (*Macropus dorsalis*) and domestic cattle. Wildl Res 19:79–87. https://doi.org/10.1071/WR9920079

Dröscher I, Kappeler PM (2014) Competition for food in a solitarily foraging folivorous primate (*Lepilemur leucopus*)?: Feeding Competition. Am J Primatol 76:842–854. https://doi.org/10.1002/ajp.22272

Eisenhofer R et al (2023) Individuality and stability of the koala (*Phascolarctos cinereus*) faecal microbiota through time. PeerJ 11: e14598. https://doi.org/10.7717/peerj.14598

Fabrice S et al (2024) Traditional uses of the plants consumed by *Propithecus deckenii* (Peters, 1870) in mandrozo protected area, Western Madagascar. Int J Prog Sci Technol 42:171–181

Farida WR (2022) Habitat and Distribution of Cuscuses (Phalangeridae). In: Rosichon U, Abinawanto D (eds) Indonesian Cuscuses (Diprotodontia: Phalangeridae): Status and Perspective. BP International, West Bengal, pp 16–35

Faulkner AL, Lehman SM (2006) Feeding patterns in a small-bodied nocturnal folivore (*Avahi laniger*) and the influence of leaf chemistry: a preliminary study. Folia Primatol (Basel) 77:218–227. https://doi.org/10.1159/000091231

Galliez M, de Souza Leite M, Queiroz TL, dos Santos Fernandez FA (2009) Ecology of the water opossum *Chironectes minimus* in Atlantic forest streams of southeastern Brazil. J Mammal 90:93–103. https://doi.org/10.1644/07-mamm-a-397.1

Ganzhorn JU (1986) Feeding behavior of *Lemur catta* and *Lemur fulvus*. Int J Primatol 7:17–30. https://doi.org/10.1007/bf02692307

Ganzhorn JU, Pietsch T, Fietz J, Gross S (2004) Selection of food and ranging behaviour in a sexually monomorphic folivorous lemur: *Lepilemur ruficaudatus.* J Zool 263:393–399. https://doi.org/10.1017/S0952836904005394

Gould L, Power ML, Ellwanger N, Rambeloarivony H (2011) Feeding behavior and nutrient intake in spiny forest-dwelling ring-tailed lemurs (*Lemur catta*) during early gestation and early to mid-lactation periods: compensating in a harsh environment. Am J Phys Anthropol 145:469–479. https://doi.org/10.1002/ajpa.21530

Grassi C (2006) Variability in habitat, diet, and social structure of *Hapalemur griseus* in Ranomafana National Park, Madagascar. Am J Phys Anthropol 131:50–63. https://doi.org/10.1002/ajpa.20423

Harcourt C (1986) Seasonal variation in the diet of South African galagos. Int J Primatol 7:491-506. https://doi.org/10.1007/BF02693660

Harcourt C (1991) Diet and behaviour of a nocturnal lemur, *Avahi laniger*, in the wild. J Zool 223:667–674. https://doi.org/10.1111/j.1469-7998.1991.tb04395.x

Harris JM, Maloney K (2010) *Petauroides volans* (Diprotodontia: Pseudocheiridae). Mamm Species 42:207–219. https://doi.org/10.1644/866.1

Heinsohn TE (2002) Observations of probable camouflaging behaviour in a semi-commensal common spotted cuscus *Spilocuscus maculatus maculatus* (Marsupialia: Phalangeridae) in New Ireland, Papua New Guinea. Aust Mammal 24:243. https://doi.org/10.1071/am02243

Hladik C, Charles-Dominique P, Petter J (1980) Feeding strategies of five nocturnal prosimians in the dry forest of the west coast of Madagascar. In: Charles-Dominique P et al. (eds) Nocturnal Malagasy Primates. Ecology, Physiology, and Behavior. Academic Press, New York, pp 41–73. https://doi.org/10.1016/B978-0-12-169350-3.50007-1

Hladik CM (1979) Diet and ecology of prosimians. In: G.A. Doyle, R.D. Martin (eds) The Study of Prosimian Behavior. Academic Press, New York, pp 307–357

Hume ID, Jazwinski E, Flannery TF (1993) Morphology and function of the digestive-tract in New Guinean possums. Aust J Zool 41:85. https://doi.org/10.1071/zo9930085

Hunsaker D II (ed) (2012) The biology of marsupials. Academic Press, San Diego

Julien-Laferrière D (1999) Foraging strategies and food partitioning in the neotropical frugivorous mammals *Caluromys philander* and *Potos flavus*. J Zool (1987) 247:71–80.

https://doi.org/10.1111/j.1469-7998.1999.tb00194.x

Latinis, D. K. 1996. Hunting the cuscus in Western Seram: the role of the phalanger in subsistence economies in Central Maluku. Cakalele 7: 17-32.

Marsh KJ, Blyton MDJ, Foley WJ, Moore BD (2021) Fundamental dietary specialisation explains differential use of resources within a koala population. Oecologia 196:795–803. https://doi.org/10.1007/s00442-021-04962-3

Martin RW (2005) Tree-kangaroos of Australia and New Guinea. CSIRO Publishing, Clayton.

McManus JJ (1974) *Didelphis virginiana*. Mammal Species 40:1–6. https://doi.org/10.2307/3503783

Medellín RA (1991) Ecomorfología del cráneo de cinco didélfidos: tendencias, divergencias e implicaciones. An Inst Biol UNAM Zool 62:269–286

Meserve P, Lang B, Patterson BD (1988) Trophic relationships of small mammals in a Chilean temperate Rainforest. J Mammal 69:721–730. https://doi.org/10.2307/1381627

Mondolfi E, Padilla GM (1958) Contribución al conocimiento del "perrito de agua" (*Chironectes minimus* Zimmermann). Mem. Soc. Cienc. Nat. "La Salle" 17:141–155

Nekaris A, Bearder SK (2007) The Lorisiform primates of Asia and mainland Africa. In: Campbell CJ, Fuentes A, MacKinnon KC, Bearder SK, Stumpf RM (eds) Primates in Perspective. Oxford University Press, Oxford, pp 24–45

Nekaris KAI, Jayewardene J (2003) Pilot study and conservation status of the slender loris (*Loris tardigradus* and *L. lydekkerianus*) in Sri Lanka. Primate Conserv 19:83–90

Overdorff DJ (1993) Similarities, differences, and seasonal patterns in the diets of *Eulemur rubriventer* and *Eulemur fulvus rufus* in the Ranomafana National Park, Madagascar. Int J Primatol 14:721–753. https://doi.org/10.1007/bf02192188

Parreira Claro HW, Hannibal W (2022) Diet of small mammals in semi-deciduous forest fragments in Central Brazil. Austral Ecol 47:770–774. https://doi.org/10.1111/aec.13169

Petter J, Schilling A, Parienté G (1971) Observations éco-éthologiques sur deux lémuriens malgaches nocturnes: *Phaner furcifer* et *Microcebus coquereli*. Rev. Ecol. (Terre Vie) 25:287–327. https://doi.org/10.3406/revec.1971.4639

Powzyk JA, Mowry CB (2003) Dietary and feeding differences between sympatric *Propithecus diadema diadema* and *Indri indri*. Int J Primatol 24:1143–1162. https://doi.org/10.1023/B:IJOP.0000005984.36518.94

Powzyk JA, Mowry CB (2006) The feeding ecology and related adaptations of *Indri indri*. In: Developments in Primatology: Progress and Prospect. Springer US, Boston, pp 353–368. https://doi.org/10.1007/978-0-387-34586-4_16

Randimbiharinirina DR et al (2018) Behaviour and ecology of male aye-ayes (*Daubentonia madagascariensis*) in the Kianjavato Classified Forest, south-eastern Madagascar. Folia Primatol 89:123–137. https://doi.org/10.1159/000486673

Randrianarison RM et al (2022) Feeding ecology and regurgitation–reingestion behavior of the critically endangered *Indri indri* in the maromizaha protected area, eastern Madagascar. Int J Primatol 43:584–610. https://doi.org/10.1007/s10764-022-00298-8

Rawlins DR, Handasyde KA (2002) The feeding ecology of the striped possum *Dactylopsila trivirgata* (Marsupialia: Petauridae) in far north Queensland, Australia. J Zool 257:195–206. https://doi.org/10.1017/S0952836902000808

Robinson JG, Redford KH (1986) Body size, diet, and population density of neotropical forest mammals. Am Nat 128:665–680. https://doi.org/10.1086/284596

Runcie MJ (2002) Behaviour and Ecology of Tropical Rock-possums: The Rock-haunting Possum, *Petropseudes dahli* and the Scaly-tailed Possum, *Wyuld Squamicaudata*. PhD thesis, Charles Darwin University, Australia. https://doi.org/10.25913/5eab8873df9f7

Saragih EW, Sadsoeitoeboen MJ, Pattiselanno F (2010) The diet of spotted cuscus (*Spilocuscus maculatus*) in natural and captivity habitat. Nusant Biosci 2:78-83. https://doi.org/10.13057/NUSBIOSCI/N020205

Schülke O (2003) To breed or not to breed? Food competition and other factors involved in female breeding decisions in the pair-living nocturnal fork-marked lemur (*Phaner furcifer*). Behav Ecol Sociobiol 55:11–21. https://doi.org/10.1007/s00265-003-0676-2

Siciliano Martina L (2013) *Lestoros inca* (Incan shrew opossum). *Animal Diversity Web*. <https://animaldiversity.org/accounts/Lestoros_inca/> (16 July 2025)

Simmen B, Hladik A, Ramasiarisoa P (2003) Food intake and dietary overlap in native *Lemur catta* and *Propithecus verreauxi* and introduced *Eulemur fulvus* at Berenty, Southern Madagascar. Int J Primatol 24:949–968. https://doi.org/10.1023/a:1026366309980

Smith AP, Russell R (1982) Diet of the yellow-bellied glider *Petaurus australis* (Marsupialia: Petauridae) in north Queensland. Aust Mammal 5:41-45 https://doi.org/10.1071/am82004

Stephens SA, Salas LA, Dierenfeld ES (2006) Bark Consumption by the Painted Ringtail (*Pseudochirulus forbesi larvatus*) in Papua New Guinea. Biotropica 38:617–624. https://doi.org/10.1111/j.1744-7429.2006.00197.x

Sterling EJ (1994) Aye-ayes: specialists on structurally defended resources. Folia Primatol 62:142–154. https://doi.org/10.1159/000156771

Streilein KE (1982) Ecology of small mammals in the semiarid Brazilian Caatinga. I. Climate and faunal composition. Ann Carnegie Mus 51:79-107. https://doi.org/10.5962/p.330738

Sussman R (1977) Feeding behaviour of *Lemur catta* and *Lemur fulvus*. In: Clutton-Brock TH (ed) Primate ecology: studies of feeding and ranging behaviour in lemurs, monkeys and apes. Academic Press Inc., London, pp 1–36

Syahrullah FN, Maddus U, Mustari A, Gursky S, Indrawan M (2023) Distribution and abundance of peleng tarsier (*Tarsius pelengensis*) in Banggai Island group, Indonesia. Sci Rep 13: 11445. https://doi.org/10.1038/s41598-023-30049-5

Thalmann U (2001) Food resource characteristics in two nocturnal lemurs with different social behavior: *Avahi occidentalis* and *Lepilemur edwardsi*. Int J Primatol 22:287–324. https://doi.org/10.1023/a:1005627732561

Tierney PJ (1985) Habitat and ecology of the bridled nailtail wallaby, *Onychogalea fraenata*, with implications for management. Master thesis, Queensland University of Technology, Australia

Tuft K, Crowther M, McArthur C (2011) Multiple scales of diet selection by brush-tailed rock-wallabies (*Petrogale penicillata*). Aust Mammal 33:169–180. https://doi.org/10.1071/AM10041

de Vries D et al (2024) Comparison of dental topography of marmosets and tamarins (Callitrichidae) to other platyrrhine primates using a novel freeware pipeline. J Mamm Evol 31. https://doi.org/10.1007/s10914-024-09704-9

Williamson GM (1986) The ecology of the dama wallaby (*Macropus eugenii*, Desmarest) in forests at Rotorua, with special reference to diet. MSc thesis, Massey University, New Zealand

Wilson DE, Mittermeier RA (eds) (2015) Handbook of Mammals of the World: 5. Monotremes and Marsupials. Lynx Edicions, Barcelona
